# Molecular cloning, phylogenetic analysis, and expression profiling of endoplasmic reticulum molecular chaperone *BiP* genes from bread wheat (*Triticum aestivum* L.)

**DOI:** 10.1186/s12870-014-0260-0

**Published:** 2014-10-01

**Authors:** Jiantang Zhu, Pengchao Hao, Guanxing Chen, Caixia Han, Xiaohui Li, Friedrich J Zeller, Sai LK Hsam, Yingkao Hu, Yueming Yan

**Affiliations:** College of Life Science, Capital Normal University, Beijing, 100048 China; Department of Plant Breeding, Center of Life and Food Sciences Weihenstephan, Technical University of Munich, Freising-Weihenstephan, D-85354 Germany

**Keywords:** Wheat, BiP, Cloning, Expression, HMW-GS silencing, Drought stress

## Abstract

**Background:**

The endoplasmic reticulum chaperone binding protein (BiP) is an important functional protein, which is involved in protein synthesis, folding assembly, and secretion. In order to study the role of BiP in the process of wheat seed development, we cloned three BiP homologous cDNA sequences in bread wheat (*Triticum aestivum*), completed by rapid amplification of cDNA ends (RACE), and examined the expression of wheat BiP in wheat tissues, particularly the relationship between BiP expression and the subunit types of HMW-GS using near-isogenic lines (NILs) of HMW-GS silencing, and under abiotic stress.

**Results:**

Sequence analysis demonstrated that all BiPs contained three highly conserved domains present in plants, animals, and microorganisms, indicating their evolutionary conservation among different biological species. Quantitative reverse transcription-polymerase chain reaction (qRT-PCR) revealed that *TaBiP* (*Triticum aestivum* BiP) expression was not organ-specific, but was predominantly localized to seed endosperm. Furthermore, immunolocalization confirmed that TaBiP was primarily located within the protein bodies (PBs) in wheat endosperm. Three *TaBiP* genes exhibited significantly down-regulated expression following high molecular weight-glutenin subunit (HMW-GS) silencing. Drought stress induced significantly up-regulated expression of *TaBiPs* in wheat roots, leaves, and developing grains.

**Conclusions:**

The high conservation of BiP sequences suggests that BiP plays the same role, or has common mechanisms, in the folding and assembly of nascent polypeptides and protein synthesis across species. The expression of *TaBiPs* in different wheat tissue and under abiotic stress indicated that *TaBiP* is most abundant in tissues with high secretory activity and with high proportions of cells undergoing division, and that the expression level of BiP is associated with the subunit types of HMW-GS and synthesis. The expression of *TaBiPs* is developmentally regulated during seed development and early seedling growth, and under various abiotic stresses.

**Electronic supplementary material:**

The online version of this article (doi:10.1186/s12870-014-0260-0) contains supplementary material, which is available to authorized users.

## Background

The endoplasmic reticulum (ER) is involved in protein synthesis and the folding, assembly, transport, and secretion of nascent proteins [1]. One of the most important functions of the ER involves the quality control of nascent proteins, which is accomplished by ER chaperone proteins such as protein disulfide isomerase (PDI) and binding protein (BiP). As one of the major ER chaperone proteins, BiP plays important roles in protein synthesis, folding, and assembly [2].

BiP belongs to the HSP70 family of chaperone proteins. It has an ATPase domain at the N terminus and a protein-binding domain at the C terminus, which allows BiP to cycle between adenosine triphosphate (ATP) hydrolysis and adenosine diphosphate (ADP) exchange, coupled to the binding and release of its unfolded protein [3,4]. The BiP protein includes a KDEL or HDEL ER retention signal at the C terminus, which functions to retain the protein in the ER lumen. In general, BiP chaperone proteins have two main functions in the ER. The first is to bind unfolded proteins that enter into the ER lumen, thereby preventing nascent polypeptide chains from folding incorrectly or polymerizing. The second function of BiP is to interact with nascent immature secretory proteins synthesized from membrane-bound polysomes in the ER. This prevents immature protein denaturation or degradation, and ensures proper folding. Thus, BiP participates not only in assisting protein folding, but also in the protein degradation process known as ER-associated degradation (ERAD). When unfolded or mis-folded proteins accumulate at high levels in the ER lumen, BiP induces ERAD to remove these abnormal proteins from the folding pathway [5].

The genes encoding BiP isolated from maize, rice, *Arabidopsis*, pumpkin, and other plants appear to be highly conserved, particularly in more closely related species [6]. The involvement of BiPs in the synthesis of high levels of storage proteins and stress responses has been reported [7-9]. BiP forms complexes with nascent chains of prolamines in polyribosomes and with free prolamines, and retains prolamines in the lumen by facilitating their folding and assembly into protein bodies (PBs) [10]. Severe suppression (*BiP1*KD) or significant over-expression (*BiP1*OEmax) of *BiP1* not only alters rice seed phenotype and the intracellular structure of endosperm cells, but also reduces seed storage protein content, starch accumulation, and grain weight [6]. This indicates that the expression levels of BiPs affect the synthesis and accumulation of seed storage proteins and starches that are related to grain quality and yield.

Various environmental factors can cause an ER stress response, including temperature, light, drought, and salt. Some studies have shown that the expression of BiP is closely related to ER stress responses. For example, a change in light intensity can cause changes in the level of BiP expression in specific tissues of *Arabidopsis*, and regulates the accumulation levels of the secreted proteins [11]. Interestingly, transgenic plants overexpressing BiP exhibited better endurance and less sensitivity to drought than the wild type. In addition, under the same drought conditions, transgenic plants overexpressing BiP exhibited higher leaf water content, reduced withering, and reduced stomatal closures compared with the wild type. In contrast, certain biological parameters related to drought in these transgenic plants, such as the contents of proline and glucose, exhibited no significant changes compared with the wild type [12]. These findings suggest that overexpression of *BiP* may shut down the expression of other drought-induced genes, and may lead to the increased tolerance of the transgenic plants compared with the wild type.

As an allohexaploid species, bread wheat (*Triticum aestivum* L., 2n = 6× = 42, AABBDD) is one of the most important and widely cultivated crops in the world. Wheat storage proteins, mainly polymeric glutenins and monomeric gliadins, primarily determine the processing quality of wheat flour by contributing to its unique visco-elastic properties for the production of bread and other food products [13]. In particular, high molecular weight glutenin subunits (HMW-GS), as important components of glutenins, play a key role in governing bread-making ability by forming large polymeric structures through disulfide bonds [14]. Studies have shown that BiP involved in the synthesis of storage proteins in wheat, including HMW-GS, and BiP accumulated to maximum level in the middle stage of endosperm development, a period of rapid cell expansion and HMW-GS accumulation [15]. Although forming a declining trend in the latter of HMW-GS accumulation, the pattern of BiP accumulation was compatible with a proposed role as catalysts for storage protein folding and accumulation in the ER, and was detected in the latter of endosperm development [16].

Although BiPs have been investigated in some plant species, their structures, phylogenetic evolution, and functional properties in wheat have remained uncertain. In this study, three homologous cDNA sequences of *BiPs* in bread wheat were cloned for the first time, and their structural features, evolutionary conservation, expression profiles in different organs, and expression under drought stress were investigated. Our results demonstrate that BiPs are highly conserved among animals, microorganisms, and plants, and that their expression levels are closely related to HMW-GS synthesis and drought tolerance. These findings provide new insights into the structures, evolution, and functions of the BiP family.

## Results

### Molecular characterization of *BiP* genes in bread wheat

The complete cDNA sequences of *TaBiPs* in bread wheat variety Chinese Spring (CS) were amplified using specific primers and an expected product of approximately 1670 bp was amplified by RACE (see Additional file 1). After cloning and sequencing, a 1665 bp sequence contained the conserved partial length of the *BiP* cDNA sequence. DNA sequence analysis identified the presence of the open reading frame (ORF), but without the coding sequences for the N- and C-terminal ends. Therefore, a PCR-based method was used to isolate the remaining 5’ and 3’ ends of the *BiP* cDNA. Finally, three complete cDNA sequences of *TaBiP* genes, named *TaBiP1*, *TaBiP2,* and *TaBiP3*, were obtained and deposited in GenBank with accession numbers KC894715, KC894716, and KC894717, respectively.

**Figure 1:**
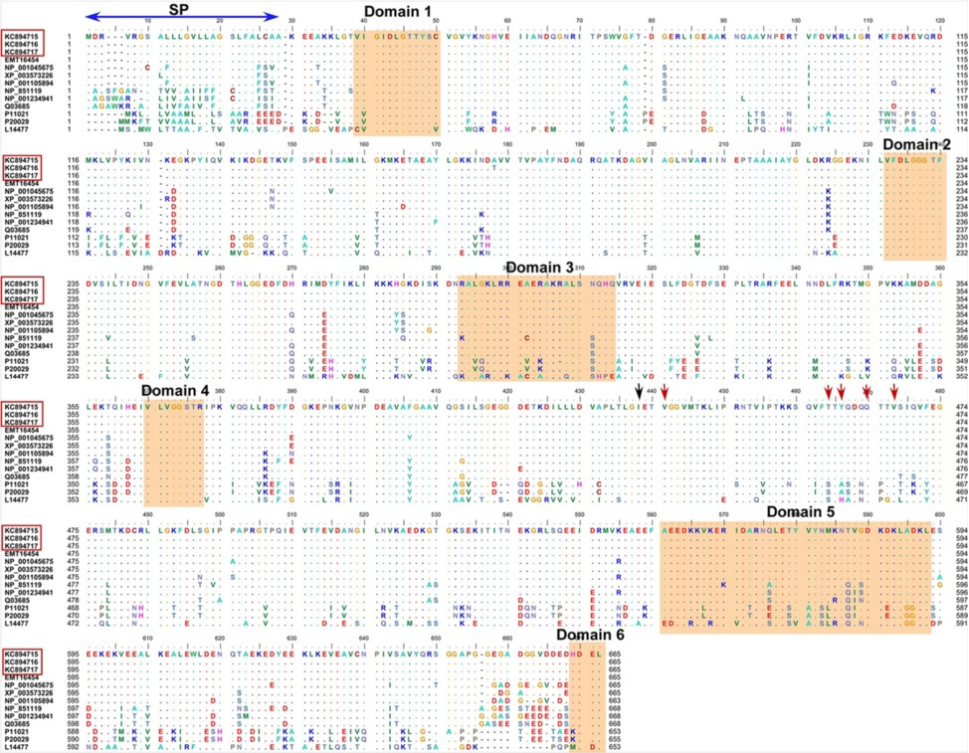
**The alignment analysis of representative BiP amino acid sequences.** The deduced amino acid sequences of wheat are KC894715, KC894716, and KC894717 (red boxes). SP, signal peptide; Domain1, β motif; Domain2, γ motif; Domain3, calmodulin-binding site; Domain4, a denosine-binding motif; Domain5, αβ motif; Domain6, ER retention signal–HDEL; blue arrow, the sequences of SP; black arrow, the GI cut-off point; red arrows, the sites of hydrogen bonds.

cDNA sequence analysis indicated that *TaBiP1*, *TaBiP2*, and *TaBiP3* had sizes of 2163, 2155, and 2158 bp, respectively, but that the coding regions of all genes consisted of a 1998 bp sequence encoding 665 amino acid residues (Figure 1). In addition, three corresponding full length genomic DNA sequences were obtained; the complete sequence lengths of *TaBiP1*, *TaBiP2*, and *TaBiP3* genes were 3725, 3701, and 3691 bp, respectively. Further, chromosomal localization studies showed that *TaBiP1* is located on chromosome 6DS, *TaBiP2* is located on chromosome 6BS, and *TaBiP3* is located on chromosome 6AS. After searching and analyzing the wheat genome sequences completed recently through WHEAT URGI, we found that each wheat genome only has one *BiP* gene, indicating that common wheat may have three BiP gene copies. All three genes comprised eight exons and seven introns that were highly conserved (see Additional file 2). The molecular characterization of the three cloned *BiP* homologous genes in wheat is shown in Table 1.

**Table 1:** **The molecular characterization of BiP genes in common wheat**

**BiP genes**	**GenBank accession no.**	**cDNA/DNA length (bp)**	**ORF length (amino acids)**	**5′UTR (bp)**	**3′UTR (bp)**	**Exon**	**PI**	**Mw (kDa)**
*TaBiP1*	KC894715	1998/3725	665	103	137	8	5.0	70.7
*TaBiP2*	KC894716	1998/3701	665	109	123	8	5.0	70.7
*TaBiP3*	KC894717	1998/3691	665	97	113	8	5.0	70.7
*BdBiP1*	XP_003573226	1998/4101	665	103	310	8	5.0	70.7
*0sBiP1*	NP_001045675	1998/3956	665	147	330	8	5.0	70.8

Alignment of the deduced TaBiP amino acid sequences with BiP homologs from other species revealed a high level of conservation among domains, although some variations were present. In particular, TaBiPs exhibited higher similarity to BiPs from maize, rice, and *Brachypodium distachyon*, including similar coding regions and ORFs as well as functional domains (Figure 1). In general, BiP proteins have an ATPase domain at the N terminus (approximately 45-kDa), which contains stretches of highly conserved sequence, an ATPase activity region, and a protein-binding domain at the C terminus [17]. The C-terminal region includes a 16-kDa segment that possesses a peptide-binding site and a more variable 10-kDa sequence comprising the terminal part of the protein [17,18]. This structure allows BiP to cycle between ATP and ADP exchange, coupled to the binding and release of unfolded proteins [3]. Both domains in the C terminus have a cut-off point GI, the cleavage site dividing the ATPase domain from the peptide-binding domain (Figure 1).

All BiPs have a signal peptide sequence at the beginning of the N terminus (Figure 1), the main function of which is to guide the membrane transport of the different BiP protein strains. The length of the signal peptide sequences differs among species. For example, the signal peptides in rice, maize, wheat, *Brachypodium*, and *Sorghum* have 24 amino acid residues (aa), whereas those in *Arabidopsis*, spinach, tobacco and soybean contain 27, 28, 29, and 30 aa, respectively. Notably, the signal peptide in Douglas fir has only 17 aa [9].

Some important motifs with different functions in the ATPase domain of BiPs are highly conserved. As shown in Figure 1, the β (Domain 1), γ (Domain 2), and adenosine-binding (Domain 4) motifs are located in the ATPase domain, and their functions are to bind ATP or release ADP. A putative calmodulin-binding motif (Domain 3) is also located in the ATPase domain [19,20].

The C-terminal protein-binding domains of BiPs has five highly conserved amino acid residues (Figure 1), which form a five-residue substrate core and facilitate hydrogen-bonding with the peptide-substrate backbones. The αβ motif (Domain 5) located in the C-terminal protein-binding domain mainly prevents the release of nascent peptide substrates from the protein-binding pocket [21]. In addition, the C terminus of BiP has a highly conserved HDEL sequence (Domain 6), which acts as an ER retention signal. However, there are some variations in the retention signal. HDEL is present in most of the plant BiPs, whereas KDEL is present in mammals, and MDDL is found in certain bacterial species (Figure 1) [9].

### Single base substitutions and insertion/deletions (InDels) in the *BiP* genes of wheat

The complete coding sequences of three cloned *TaBiP* genes were aligned with 11 *BiP* genes from other cereal crops (*O. sativa* BiP1/2 from rice, *Z. mays* BiP1/2 from maize, *B. distachyon* BiP1/2 from *B. distachyon*, *S. bicolor* BiP1/2 from *Sorghum*, and *S. italica* BiP1/2/3 from *Setaria italica*) to detect single base substitution and InDels. A total of 14 single base substitutions were identified at different positions, the number of substitutions in *TaBiP1*, *TaBiP2*, and *TaBiP3* being 8, 5, and 4, respectively (Table 2). However, no InDels were found. Of the 14 single base substitutions detected, 11 (70%) were the result of transitions (A–G or C–T), and only three substitutions were attributed to transversions (A–T, A–C, C–G, or G–T). Six substitutions at positions 72, 96, 228, 252, 834, and 1404 involved non-synonymous changes that could lead to amino acid substitutions. The remaining eight single base substitutions involved synonymous substitutions that did not cause amino acid changes.

**Table 2:** **Positions of single base substitutions identified in the three cloned BiP homologs**

***BiP*** **genes**	**72**	**96**	**141**	**165**	**228**	**252**	**514**	**834**	**942**	**1012**	**1404**	**1530**	**1581**	**1698**
*TaBiP1*	T	C	**T**	C	**G**	**C**	G	**T**	G	**T**	**T**	**T**	**T**	C
*TaBiP2*	**C**	C	C	C	C	A	**A**	**T**	**A**	C	C	C	G	**T**
*TaBiP3*	T	**T**	**T**	**T**	**G**	A	G	C	G	C	C	C	G	C
Other 11*BiP* genes	T	C	C	C	C	A	G	C	G	C	C	C	G	C

Single base substitutions are indicated in boldface. The other 11 BiP genes are *O. sativa BiP1* (GENBANK: NP_001045675); *O. sativa BiP2* (NP_001055339); *B. diumdistachyon BiP1* (XP_003573226); *B. diumdistachyon BiP2* (XP_003565461); *Z. mays BiP1* (U56208); *Z. mays BiP2* (U56209); *S. bicolor BiP1* (XM_002456746); *S. bicolor BiP2* (XM_004971841); *S. italica BiP1* (XP_004971898); *S. italica BiP2* (XP_004971892); *S. italica BiP3* (XP_004964075).

### Phylogenetic and conserved motif analysis of BiPs among different species and prediction of TaBiP tertiary structure

**Figure 2:**
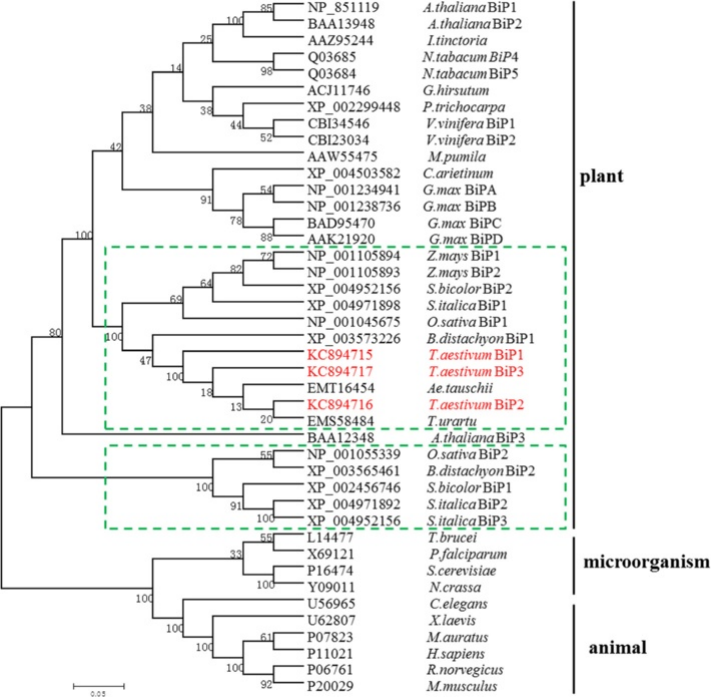
**A phylogenetic tree of a representative sampling of BiP amino acid sequences.** Amino acid sequences and accession numbers are provided in Methods. The TaBiPs are shown in red font.

Forty-two BiP amino acid sequences were used to construct an unrooted phylogenetic tree according to Zhu et al [22], for analysis of the evolutionary relationships among different species, including three from *T. aestivum*, two from *O. sativa*, two from *Z. mays*, two from *B. distachyon*, and the 33 sequences from other species. The resulting phylogenetic tree clearly differentiated the proteins into three branches, corresponding to plants, animals, and microorganisms, indicating greater divergence of BiPs between different biological species during long-term evolutionary processes, as well as formation of a distinct phylogenetic plant subgroup (Figure 2). Among the plant BiPs, the phylogenetic tree was divided into several separated small subgroups, including species of the Leguminosae and Poaceae families. Two closely related subfamilies were also identified within the Poaceae, marked with green boxes in Figure 2.

Analysis of the conserved motifs of BiPs from different biological species demonstrated that all BiPs contain three motifs (see Additional file 3), that are highly conserved in both position and length, with only minor variations. Motif 3 contained a β motif (Domain 1), motif 2 included a γ motif (Domain 2), and motif 1 is the region where hydrogen-bonding occurs. The β and γ motifs belong to the ATPase domain, whereas motif 3 is part of the C-terminal protein-binding domain.

**Figure 3:**
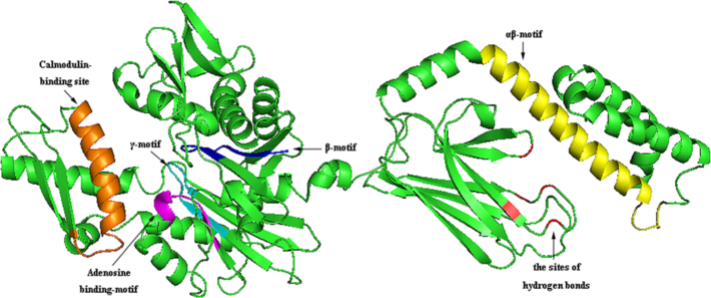
**The tertiary structure of TaBiP protein.** The protein structure was rendered using the PyMol 2 server, and appeared similar in structure to that of the plant HSP70 proteins predicted by Sung et al. [23]. The β, γ, adenosine-binding motifs, and the calmodulin-binding motif are located in the N-terminal ATPase domain, and are color-coded in blue, cyan, magenta, and orange, respectively. The αβ motif (yellow) and five binding sites of hydrogen-bonds (red) are located in the C-terminal domain.

Since BiP is a member of the HSP70 family, the tertiary structure of BiP should be similar to that of HSP70 proteins. Indeed, the predicted tertiary structure of cloned homologous BiP constructed using Pymol.2 (Figure 3) was very similar to that of HSP70 proteins [23]. These motifs occupy similar relative positions within the tertiary structures of BiPs from different species, as seen in Figure 3. The ATP-binding site in the N-terminal domain is situated at the base of a deep cleft positioned between two structural lobes. Surprisingly, the nucleotide-binding “core” of the ATPase domain was found to have a tertiary structure similar to that of hexokinase [24], suggesting that the phosphate transferase mechanisms and substrate-induced conformational changes of the two proteins may be similar. The peptide-binding domain is similar to those of *E. coli* DnaK, and forms a β-sandwich peptide-binding pocket where the peptide-binding cleft is located (Figure 3). The residues lining the cleft interact with hydrophobic stretches of unfolded and exposed polypeptide chains (Figure 3). A C-terminal α-helical extension serves as a lid to trap a peptide bound in the binding cleft, thereby providing a mechanism for maintaining long-lived complexes [21].

### Expression profile of *TaBiP* genes in different wheat organs

**Figure 4:**
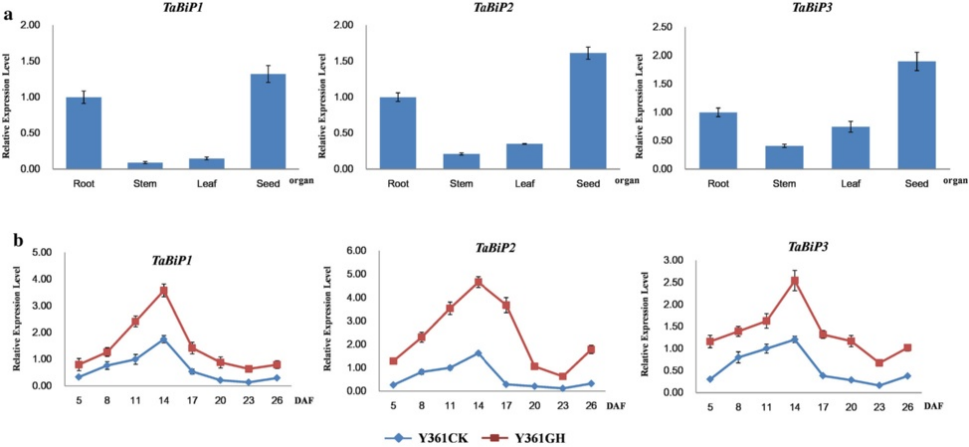
**qRT-PCR analysis of** ***TaBiP*** **transcriptional expression in different wheat organs, developing seeds, and under drought stress. (a)** Expression in wheat organs. **(b)** Expression in developing seeds and under drought stress. Yanyou 361 CK (Y361CK) was not treated, and Yanyou 361 GH (Y361GH) was drought-treated.

Expression profiles of the three obtained *TaBiP* genes in the roots, stems, leaves, and seeds of wheat were investigated by qRT-PCR (Figure 4a). The results indicated that all three *TaBiP* genes are expressed in wheat roots, stems, leaves, and seeds, although the expression levels varied substantially. Apparently, the expression levels of the *TaBiPs* appeared to be high in seeds and low in both stems and leaves.

### Dynamic expression profiles of *TaBiP* genes in developing grains and under drought stress

The dynamic expression profiles of the three *TaBiP* genes during the eight grain developmental stages and under drought stress in the bread wheat cultivar Y361 exhibited an up-down expression profile during grain development (Figure 4b). The highest expression level of *TaBiP*s occurred at 14 DAF of seed development, which may be related to the rapid synthesis and accumulation stages of wheat storage proteins from 15 to 25 DAF [25]. Under drought stress, all three *TaBiP* genes displayed significant up-regulation of expression compared to the control, with the highest expression level occurring at 14 DAF (Figure 4b).

### Relationships between *TaBiP* expression and HMW-GS synthesis during grain development

**Figure 5:**
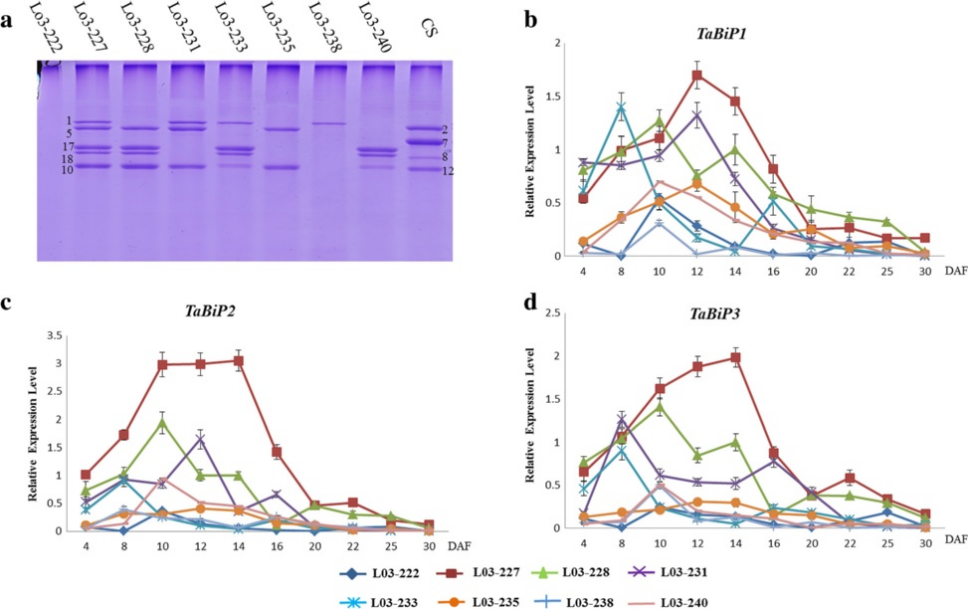
**HMW-GS compositions and dynamic transcriptional expression profiles of three** ***TaBiP*** **genes in a set of NILs as revealed by qRT-PCR. (a)** HMW-GS compositions in the NILs by SDS-PAGE. **(b–d)** Expression of *TaBiP1,* -*2,* and *-3* in developing NILs wheat seeds.

A set of HMW-GS NILs were used to define the relationships between *TaBiP* expression and HMW-GS synthesis during grain development (Table 3). SDS-PAGE analysis showed that eight NILs had different HMW-GS compositions, in which the *Glu-A1*, *Glu-B1*, and *Glu-D1* loci were silenced, and notably all HMW-GS genes were silent in L03-222 (Figure 5a). Analysis by qRT-PCR revealed significantly different *TaBiP* expression profiles corresponding to various HMW-GS silencing in different NILs (Figure 5b–d). In general, *TaBiP* genes displayed an up- to down-regulated expression pattern during grain development, with higher expression levels occurring at 10–14 DAF. All three *TaBiP* genes appeared to exhibit significantly down-regulated expression concomitant with HMW-GS silencing, with the lowest *TaBiP* expression level occurring in L03-222, in which all HMW-GS loci were silent (Figure 5b–d). These results demonstrated a close relationship between *TaBiP* expression and the subunits type of HMW-GS during grain development.

**Table 3:** **Compositions of HMW-GS in the NILs**

**NILs**	***Glu-A1***	***Glu-B1***	***Glu-D1***
L03-222	Null	Null	Null
L03-227	1	17 + 18	5 + 10
L03-228	Null	17 + 18	5 + 10
L03-231	1	Null	5 + 10
L03-233	1	17 + 18	Null
L03-235	Null	Null	5 + 10
L03-238	1	Null	Null
L03-240	Null	17 + 18	Null

### Identification of TaBiPs in wheat endosperm tissue by transmission and immuno-electron microscopy

**Figure 6:**
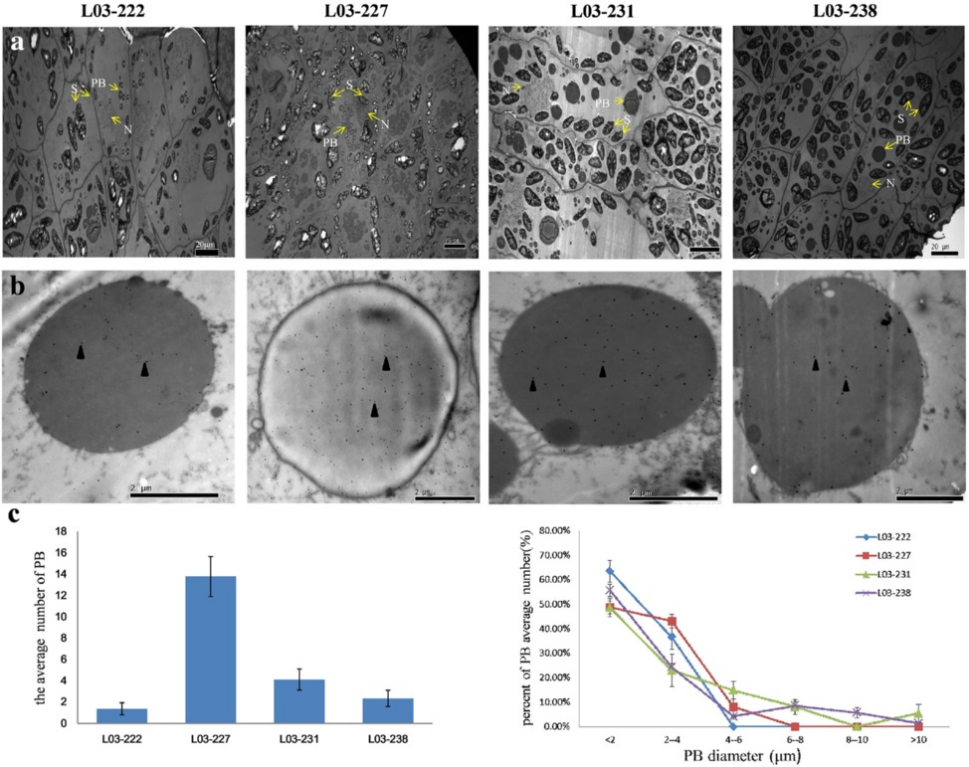
**Electron micrographs of wheat seed endosperm cells at 14 DAF in L03-222, L03-227, L03-231, and L03-238. (a)** The PB graph in one cell using transmission electron microscopy. **(b)** Ultrathin sections of wheat endosperm demonstrating the immunolocalization of BiP to PBs (black arrowhead). **(c)** The average number of PBs (left) and percentage of average numbers of different PB diameters (right). PB, protein body; N, nucleus; S, starch.

In order to clearly define the location of TaBiPs and the relationship between BiP and PBs in grain endosperm, ultrathin sections of developing wheat grain endosperm (14 DAF) from four NILs were observed by transmission electron microscopy (Figure 6a) and immuno-electron microscopy (Figure 6b). The results indicated that only small amounts and of smaller sized PBs were present in L03-222, which had no HMW-GS expression (Figure 6a). A larger number of PBs could be observed in NILs containing one or two HMW-GS (L03-231 or L03-238 in Figure 6a) compared with L02-222, and the highest numbers of PBs were observed in L03-227 with normal HMW-GS expression. Immuno-electron microscopy showed that the anti-BiP probe was primarily located at the periphery of the PBs and was observed at all stages of PB development. It is evident that the amount of anti-BiP in PBs increased with the increasing number of HMW-GS (Figure 6b). The trend observed within these results indicated that the average number of PBs (Figure 6c left) and the percentage of larger diameter PBs (Figure 6c right) increased with the increasing number of HMW-GS.

### Expression patterns of *TaBiP* genes in wheat seedlings under drought stress

**Figure 7:**
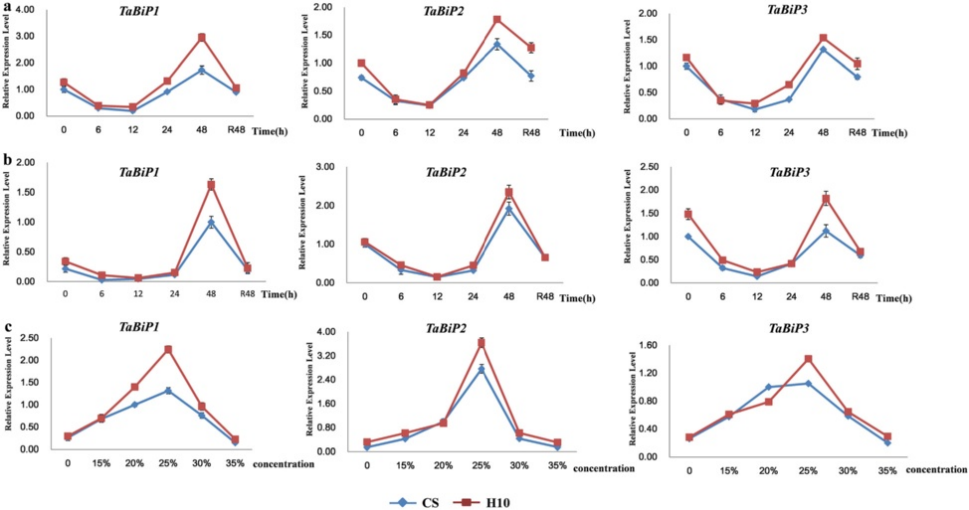
**Analysis of** ***TaBiP*** **expression in wheat seedlings under drought stress. (a)** *TaBiP* expression in the roots under 20% PEG. **(b)** *TaBiP* expression in the leaves under 20% PEG. **(c)** *TaBiP* expression in the leaves under different PEG concentrations. R48, recover 48 hours.

The expression patterns of *TaBiPs* in seedling roots and leaves under drought stress at different times and with different concentrations of PEG6000 indicated that the expression of *TaBiPs* could be regulated by drought stress. The results presented in Figure 7 show that PEG6000 treatment induced significantly up-regulated expression of *TaBiP* genes in the seedlings of CS and H10. In general, the three *TaBiP* genes displayed similar expression patterns in seedling roots and leaves subjected to different treatment times and different concentrations of PEG6000. As seen in Figure 7a–b, the genes were significantly up-regulated in both roots and leaves from 6 to 48 h after treatment with 20% PEG6000. At PEG6000 concentrations less 20% expression was significantly down-regulated from 0 to 12 h, and then up- regulated from 12 to 48 h, reaching to levels similar to the control at recovery after 48 h (Figure 7a, b). Under different PEG6000 concentrations in both cultivars, expression of the three *TaBiP* genes was increased with increasing concentration in the range 15–30% PEG, with 25% PEG inducing maximum expression. With 35% PEG, however, there was no significant effect, and seedlings grew slowly and became severely withered (Figure 7c).

## Discussion

### Evolutionary conservation and variation of *BiP* genes among different biological species

In the current study, three *BiP* cDNA and DNA sequences from wheat endosperm tissue obtained by RACE and PCR, which exhibited high sequence identity, were isolated. The three BiP genes, *TaBiP1*, *TaBiP2,* and *TaBiP3*, are located on the chromosomes 6DS, 6BS, and 6AS, respectively. The deduced BiP proteins and other BiP homologs were highly conserved with respect to functional domains and tertiary structures (Figures 1 and 3), suggesting the conserved protein function of BiP from different species. The clearest evidence of conservation is found in the motifs (see Additional file 3) of the ATPase and peptide-binding domains (Figure 1). Conserved regions important for N-terminal ATPase activity have been identified in mammalian BiP and HSP70, as well as in functionally diverse proteins such as actin and several sugar kinases [19,26,27]. This conservation indicates that the ATPase and peptide-binding domains are necessary for the survival of different biological species. Interestingly, a putative calmodulin-binding motif is present in the ATPase domain, although calmodulin has not been found in the ER lumen, suggesting that BiP may act with Ca^2+^-binding proteins to jointly modulate the function and activity of BiP.

The major differences in the BiP sequences of different species are observed mainly in the introns (Table 1; Additional file 2) and single base substitutions (Table 2), although these differences involve few amino acid changes. Although the motifs are highly conserved, there are differences in the BiP sequence lengths between different species (see Additional file 3), which may be due to segmental duplications or InDels. In tobacco [28], soybean [29,30], *Arabidopsis* [31], and maize [32], BiPs are encoded by a multigene family. According to the wheat genome information available so far (http://wheat-urgi.versailles.inra.fr/Projects), common wheat genome may have three *BiP* genes. The distinct grouping of TaBiPs differs from that of other BiPs, indicating that TaBiPs have diverged significantly from their ancestors despite major areas of sequence conservation. A total of 14 single base substitutions were identified at different positions, and of these, six were non-synonymous mutations, which did not appear to alter the function of TaBiP, although the associated structural changes have led to different classifications in cereal crops. However, a significant difference was observed in the N-terminal signal peptide, which exhibited little conservation of sequence length and identity, and this difference is very common in different species [33]. Another obvious difference, potentially due to species evolution, was observed in the C-terminal retention signal which facilitates the return of BiP to the ER after the completion of peptide chain folding and assembly, and the difference may function as a marker that can be used to distinguish different species.

### *TaBiP* expression and HMW-GS synthesis

Plant BiP proteins have been found to be most abundant in tissues with high secretory activity and high proportions of cells undergoing division [34]. Using in situ hybridization, Muench et al. [9] found a single intense band of BiP in rice endosperm tissue, but no hybridization was visible in root and leaf tissue, even following longer exposures. The absence of an observable hybridization signal suggests that BiP is expressed at a level below the detection limits of the analyses. In the present study, qRT-PCR revealed that *TaBiPs* have no organ-specific expression, but are predominantly expressed in seeds (Figure 4a). Consistent with its functions, the synthesis of BiP is induced by physiological stress conditions that promote accumulation of proteins in the ER [2,35]. BiP participates in the import, folding, and assembly of storage proteins in the ER, and may be essential for posttranslational processing of storage proteins [36]. BiP accumulated to maximal levels in the middle stage of endosperm development, and decreased at the time of maximum storage protein accumulation [37]. When protein genes are highly expressed as storage or secretory proteins, synthesis of ER-resident chaperone proteins increases to assist with the folding and assembly of these proteins [38]. The results of immunolocalization of TaBiP in wheat endosperm tissue demonstrated that TaBiP is primarily located within the PBs in wheat endosperm (Figure 6b). Moreover, the results also indicated that TaBiPs are expressed at all stages of PB development, suggesting that the expression level of TaBiP is associated with the activity level of protein synthesis. Furthermore, the immunolocalization of BiP in rice and maize is different from that in wheat. Previous research has shown that BiPs are mainly expressed at the periphery of the PB and are easily observed in the rice endosperm [10]. In contrast, BiPs probed with the same BiP antisera were not detected by immunocytochemistry in normal developing maize endosperm [39,40]. These differences of immunolocalization in rice, maize, and wheat may be caused by different processes associated with their folding.

The highest expression of BiP was found to occur during the early stage of seed development, generally at approximately 11 DAF (Figure 4b), whereas a major increase in protein synthesis and protein folding occurred at approximately 15–25 DAF [25]. In the early stages of seed development, protein synthesis is relatively low. With the development of seed, gluten and other proteins are synthesized, and subsequently folded after transport. The process of assembly, transport, and folding appears to require additional BiP proteins. However, the essential role of BiPs in the folding and assembly of prolamine would necessitate that the abundance of BiP should be similar to that of prolamine during development, thus indicating that either the BiP poly-peptide chain is very stable, or that BiP mRNA is translated more efficiently in the latter periods of seed development. Although BiP formed a declining percentage of total protein when storage protein accumulated, its pattern of accumulation was compatible with a chaperone role for storage protein folding and accumulation in the ER [37].

Different subunits affect the size, number, and structure of PBs, ultimately affecting the quality of wheat processing [41,42]. PBs form glutenin macropolymers (GMPs) by merging with each other. The presence of glutenin particles in GMPs is directly related to the presence of certain HMW-GS, and the amount of GMP increases with the increasing number of HMW-GS [43]. This suggests that the number of HMW-GS may affect the size and number of PBs, thereby affecting the merging of PBs to influence the GMPs. TEM of wheat endosperm tissue demonstrated that the amount of PBs increased with an increasing number of HMW-GS (Figure 6a and c). More HMW-GS means that peptide chain synthesis was more active during seed development, and the molecular chaperones (i.e., BiP and PDI) that play important roles in the process of protein synthesis, also exhibited a corresponding increases [17,44]. Previous studies have demonstrated that overexpressing chaperone proteins can result in improved folding and secretion efficiency and increased accumulation of foreign proteins in tobacco [45], yeast [46,47], insect cells, and mammalian cells [48]. A study of the relationship between chaperones and seed storage protein (SSP) synthesis [6], indicated that SSP levels may be increased by alleviating the ER stress, which is caused by synthesis of high amounts of SSPs, under conditions where the levels of chaperones such as BiP, CNX, and PDIL in the ER lumen are sufficient. They further demonstrated that a slightly higher level of BiP in rice seeds might have favorable effects on SSP accumulation in the presence of other chaperones, thus suggesting that BiP acts as key factor for facilitating the biosynthesis of storage proteins. In the present study, the expression of *TaBiP* in NILs (Figure 5b–d) and results of the immune electron microscopic analysis of TaBiP in wheat endosperm tissue (Figure 6b) suggested that the expression level of *TaBiP* is closely related to the amount of HMW-GS, as it increased with increasing numbers of HMW-GS. More HMW-GS means that the ER stress is stronger, and thus based on the above results, we hypothesized the following mechanism to explain the relationship between BiP expression and the synthesis of HMW-GS in seed endosperm. Although the expression of BiP is relatively stable in most tissues under normal conditions, it increases with tissue-specific synthesis of the protein in seeds, which causes the ER to produce physiological pressure. The expression of BiP is subsequently induced in order to alleviate the ER stress. Consequently, increasing the number of HMW-GS subunits leads to increased ER stress, thus inducing higher expression of BiP.

### BiP expression and diverse plant defenses

The expression of BiP may be induced by various stress conditions that also induce ER stress [2,35]. Although *BiP* is constitutively expressed under normal growth conditions, expression of some *BiP* genes is triggered in response to ER stress conditions arising from increased levels of unfolded or abnormal proteins due to high temperature exposure or treatment with the reducing agent, dithiothreitol (DTT) or an inhibitor of protein glycosylation (tunicamycin) [49-51]. Buzeli et al (2002) identified two *cis*-regulatory functional domains that are important for the spatially-regulated activation of BiP expression under normal plant development by promoter deletion analyses [52]. Noh et al (2003) also found that the expression of BiP genes in *A.thaliana* appeared to be regulated by *cis*-regulatory functional domains, which are ERSE and UPRE [53]. These results suggest that the expression of BiP genes is induced by ER stress or other stress response. The relationship between the expression of *BiP* and the ER stress response has been studied in both animals and yeast. In animal cells, the ER stress response comprises at least three distinct intracellular signal transduction pathways: an abnormal protein refolding and degradation system [54], inhibition of translation [43,55,56], and activation of the apoptosis pathway [57,58]. BiP is closely associated with the above pathways as an on/off switch or as a master regulator of ER stress sensing, through binding to and release from each related protein.

A number of studies have also investigated the relationship between *BiP* expression and the ER stress response caused by stresses in plants. In spinach, BiP was up-regulated by temperature reduction and was increasingly associated with non-native proteins following exposure of plants to low, non-freezing temperatures [59,60]. Exposure of plants to low temperature has also been shown to stimulate production of extracellular proteins believed to be necessary for survival at low temperature [61,62]. Hurkman et al. [63] studied the impact of temperature on the mRNA of BiP and protein accumulation levels, and found that when wheat was exposed to temperatures of either 37°C or 40°C, the accumulation levels of the protein and BiP mRNA varied with the different growth periods of the seed. Like mammalian cells, plant cells have evolved at least three different mechanisms that mediate ER stress: (1) transcriptional induction of genes encoding chaperones and vesicle trafficking proteins, involving either the bZIP-type or ATF6 transcription factor; (2) attenuation of genes that encode secretory proteins and induction of genes encoding anti-stresses, regulated by PEPK and ATF4 homologous proteins; and (3) up-regulation of the ERAD system for eliminating unfolded proteins in the ER, regulated by IRE1, kinase, and XBP1. The molecular mechanisms underlying the relationship between BiP and the ER stress response in plants have been characterized in *Arabidopsis* and rice. Although *Arabidopsis* and rice have genes structurally similar to *ATF6* and *IRE1* from yeast and humans, other candidate genes corresponding to *XBP1* and *PERK* have not been identified in plants. Recently, microarray hybridization experiments have revealed several unfolded protein response (UPR) target genes in *Arabidopsis* involved in ER and secretory pathway functions [64]. In *Arabidopsis*, some *BiP* genes are directly controlled by a bZIP transcription factor, AtbZIP60, which has a transmembrane domain (TMD) and is equivalent to the *ATF6* gene that is implicated in ER stress responses in rice [49-51]. When the ER is not under ER stress, ATF6 and AtbZIP60 are localized in the ER lumen through a TMD in the C-terminal region that interacts with BiP. When stress is detected in the ER, the C-terminal TMD is cleaved in the Golgi apparatus, and the cytoplasmic N-terminal activation domain containing a leucine zipper is transferred to the nucleus, where it is involved in the expression of some ER stress-related chaperone genes through binding to the ER stress response element (ERSE) *cis-element* (CCAAT-N9-CCACG) and the ERSE-II *cis-element* (ATTGG-N-CCACG) in their promoter regions. Thus, the protein encoded by this gene may be responsible for regulating the expression of chaperone genes, including BiP.

### A putative pathway of BiPs involved in protein synthesis and diverse defense responses

**Figure 8:**
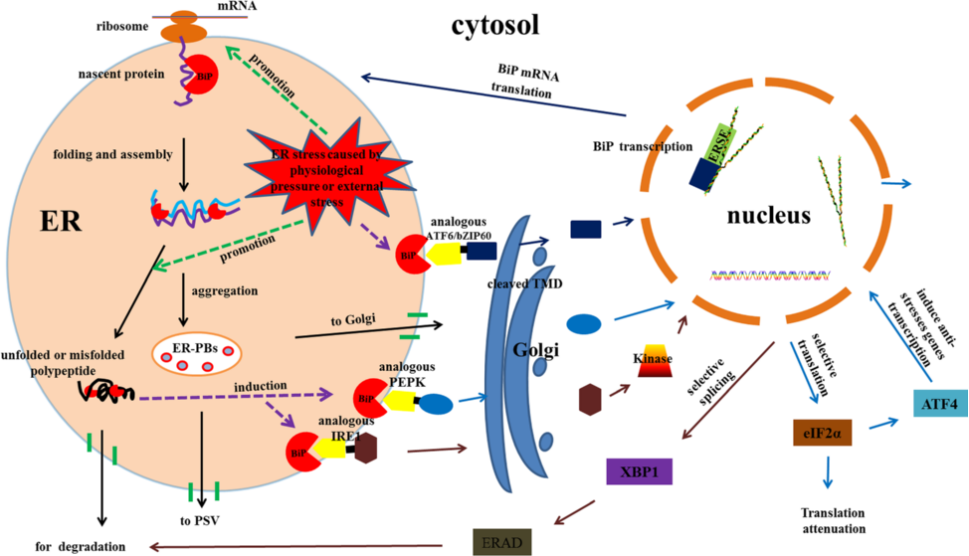
**The mechanism and signaling pathways of BiPs involved in protein synthesis and diverse defense responses.** BiP binds to nascent protein peptides, to prevent degradation or misfolding. Peptides are folded and assembled, form ER protein bodies (ER-PB), and are transported out of the ER with assistance from BiP. Misfolded or unfolded proteins are transported to the cytosol for degradation with the help of BiP. Under non-stressed conditions, BiP binds to the lumenal domains of analogous IRE1 to prevent their dimerization. When the ER is stressed, analogous ATF6 or bZiP60 released from BiP is transported to the Golgi compartment, where cleavage by TMD yields a cytosolic fragment that migrates to the nucleus to further activate transcription of UPR-responsive genes. Similarly, IRE1 released from BiP dimerizes to activate its kinase and RNAase activities to initiate XBP1 mRNA splicing, thereby creating a potent transcriptional activator to induce the gene encoding functions for ERAD. Finally, analogous PERK released from BiP dimerizes, and activates eIF2α, which leads to general attenuation of translational initiation, while eIF2α phosphorylation induces translation of ATF4 mRNA. The PERK/eIF2α/ATF4 regulatory axis thus induces expression of anti-stress response genes.

On the basis of our results and previous studies, we propose a putative pathway for BiPs involved in protein synthesis and diverse defense responses (Figure 8). Normally, BiP in the seed or other organs binds to nascent protein peptides to prevent degradation or misfolding. Subsequently, BiP helps peptides to fold and assemble in the ER, and forms the ER protein body (ER-PB). ER-PBs are transported out of the ER through different pathways, either to the Golgi or to the protein storage vacuole (PSV). Misfolded or unfolded proteins are transported to the cytosol and degraded with the assistance of BiP (Figure 8). Under non-stressed conditions, BiP binds to the lumenal domains of analogous ATF6, PEPK, and IRE1 to prevent their dimerization. Following accumulation of unfolded proteins, caused by stresses in the ER lumen, three different signal pathways would be induced and activated to relieve the ER stress: (1) transcriptional induction of genes encoding chaperones, involving the release of analogous ATF6 or bZIP60 from BiP, and their transport to the Golgi compartment where cleavage of the TMD yields a cytosolic fragment that migrates to the nucleus to further activate transcription of ER chaperone genes, including BiP; (2) activation of the ERAD system, where analogous IRE1 released from BiP dimerizes to activate its kinase and RNAase activities initiate analogous XBP1 mRNA splicing, thereby creating a potent transcriptional activator to induce genes responsible for encoding the ERAD functions; (3) induction of genes encoding anti-stressors and attenuation of genes that encode secretory proteins, analogous to PERK in plant cells that are released from BiP dimers, which activate eIF2α, leading to general attenuation of translational initiation. In addition, eIF2α phosphorylation induces ATF4. The PERK/eIF2α/ATF4 regulatory axis induces expression of anti-stress response genes [65], as seen in Figure 8.

## Conclusion

In this study, we cloned for the first time three complete *TaBiP* genes, which are all highly homologous to BiP genes in other species, suggesting that BiPs across different species share common mechanisms related to protein folding, assembly, and synthesis as well as diverse defense responses. *TaBiPs* were abundantly expressed in developing grains and are strongly associated with HMW-GS synthesis, indicating that they play critical roles in the synthesis of storage proteins and gluten quality conformation. Drought stress can induce significant up-regulation of *TaBiPs* in both seedling growth and grain development, indicating that they also perform defensive functions.

## Methods

### Plant materials

The wheat materials used in this study included the Chinese elite bread wheat cultivars Yanyou 361 (Y361) and Hanxuan 10 (H10), both of which are known for high yield, superior quality, and strong resistance to drought stress. A set of complete HMW-GS near-isogenic lines (NILs), as listed in Table 3, was kindly provided by Dr. Wujun Ma, State Agriculture Biotechnology Centre, Murdoch University, Australia. Chinese Spring (CS) was used as the control for drought stress and HMW-GS identification.

### Field planting and sampling

All materials were planted in the experimental station of the Chinese Agricultural University, Wuqiao, during the 2012–2013 growing season, using local field cultivation conditions. Each cultivar and NIL was planted in a 12-m^2^ plot with three replications and 10 rows (300 plants), respectively. The experimental site is located at longitude 116°37’23”E and latitude 37°41’02”N, and is characterized by an average of 2690 h of sunshine annually, average annual temperature of 12.6°C, and annual rainfall of 124.8 mm during the wheat season. Prior to sowing, the soil was fertilized with 200 kg/hm^2^ of urea, 400 kg/hm^2^ phosphate diamine (P_2_O_5_ 16%), 150 kg/hm^2^ K_2_SO_4,_ and 15 kg/hm^2^ ZnSO_4_. The control and treatment groups were randomized based on a block design of three replications with a seeding rate of 22.5 kg/hm^2^ and spacing of 20 cm.

The developing grains from the middle parts of the spikes in bread wheat cultivars were collected at 5, 8, 11, 14, 17, 20, 23, and 26 days after flowering (DAF), whereas the developing grains from NILs were collected at 4, 8, 10, 12, 14, 16, 20, 22, 25, and 30 DAF. All collected materials were rapidly frozen in liquid nitrogen and stored at −80°C prior to use.

### Field water deficit treatments and soil water measurement

The cultivar Y361 was subjected to water deficit treatment during the growing season. The well-watered group was normally watered with approximately 750 m^3^/hm^2^ during the sowing, jointing, and flowering stages, whereas the drought-treated group was not watered during the growth season. Soil samples of the well-watered and drought treatments with three biological replicates were taken from experimental plots at a depth of 20 cm from the top soil. The soil samples were collected in aluminum boxes and dried in an oven at 105°C for 48 h. Each sample was measured three times and the mean was used for further analysis. The soil water content (W %) was calculated using the formula: W % = (g2 − g1)/(g1 − g0) × 100% (where g2 represents the weight of the moist soil; g1 represents the weight of the dry soil; and g0 represents the weight of the empty box).

### Seedling cultivation and PEG treatments

The seeds from CS and H10 were washed using 70% alcohol followed by three washes with distilled water. Thereafter, these seeds were germinated on wet filter paper at room temperature in darkness for 24 h and transferred to the dedicated cultivate basket with full-strength Hoagland's nutrient solution containing 5 mM KNO_3_, 5 mM Ca(NO_3_)_2_, 2 mM MgSO_4_, 1mM KH_2_PO_4_, 50 μM FeNa_2_(EDTA)_2_, 50 μM H_3_BO_3_, 10 μM MnC1_2_, 0.8 μM ZnSO_4_, 0.4 μM CuSO_4_ and 0.02 μM (NH_4_)_6_MoO_24_. The nutrient solution was changed every 3 days. Drought stress analysis was conducted on seedlings, starting from the three-leaf stage by adding PEG6000 to the Hoagland’s solution. The concentrations of PEG treatment were 0% (CK), 15%, 20%, 25%, 30%, and 35%. The leaves and roots from the 20% PEG-treated group were collected at 6, 12, 24, 48, and R48 h, whereas the leaves from different PEG concentration-treated groups were collected at 48 h after treatment. All materials were immediately frozen in liquid nitrogen after harvesting and maintained at −80°C prior to RNA isolation.

### mRNA extraction, cDNA synthesis, and rapid amplification of cDNA ends (RACE)

Total RNAs were isolated based on a previously reported protocol [66]. A 1-μL RNA sample was measured using a NanoDrop ND-1000 spectrophotometer (NanoDrop Technologies, Wilmington, DE, USA) to verify the concentration and quality. The purified and non-degraded RNAs were used to synthesize cDNA with OligdT and random primers from approximately 100 ng mRNA using a superscript first-strand synthesis kit (Promega Madison, WI, USA). Primers for isolating the initial partial BiP cDNA are designed on both ends of highly conservative sequence by alignment analysis the BiP gene sequences of *O. sativa*, *Z. mays*, and *B. distachyon*. Only partial BiP cDNA clones were isolated, and therefore 5’ and 3’ RACE polymerase chain reaction (PCR) was used to obtain the coding regions, and large portions of the 5’ and 3’ untranslated regions (UTRs). The 5’ and 3’ - Full RACE Kit was obtained from TaKaRa Biotech. The specific primers for cDNA and DNA cloning were designed using Primer Premier 5.0 software, and their amplification products, separated by 1% agarose gel electrophoresis, are presented in Additional file 1. The amplicon fragments were purified from gels by using the Gel Extraction Kit (Omega), ligated into the pGEM-T Easy vector (Tiangen, Beijing, China), and then transferred into competent cells of *Escherichia coli* DH-5α strain. The sequencing of cloning products was performed by Sangon Biotech Co. Ltd., Shanghai, China.

### Sequences alignment and chromosomal localization, and identification of single base substitutions and insertion/deletions (InDels)

Sequence alignment was completed using ClustalX 1.81 software. The chromosomal localization was analyzed through the WHEAT URGI (http://wheat-urgi.versailles.inra.fr/Projects). The identification of single base substitutions and InDels among BiP genes from *Triticum* and other cereal species was based on multiple sequence alignments performed using Bioedit 7.0 software.

### Phylogenetic and conserved motif analysis of BiP family proteins and prediction of TaBiP tertiary structure

The cloned BiP sequences, together with those from different species identified through the National Center for Biotechnology Information (NCBI: http://www.ncbi.nlm.nih.gov/), SWISS-PORT (http://cn.expasy.org/sprot), EMBL (http://www.ebi.ac.uk/), and Phytozome v9.1 (http://www.phytozome.net) databases, were used to construct a phylogenetic tree with MEGA software 5.10 using the neighbor-joining (NJ) method and 1,000 bootstrap replicates. The all amino acid sequences are included within the Additional file 4. The BiP amino acid sequences of the entire coding regions were aligned using ClustalX parameters. The conserved motifs were identified and located by using MEME (http://meme.sdsc.edu/meme4_3_0/intro.html). Prediction of the tertiary structure of wheat BiP was completed using the PyMol 2 server.

### Immunolocalization of TaBiP in wheat endosperm tissue using transmission electron microscopy (TEM)

Fixation, embedding, sectioning, immunostaining, and TEM observation of the developing seeds of NIL L03-222, L03-227, L03-231, and L03-238 at 14 DAF were performed according to previously reported methods [6]. The primary antibody used was maize anti-BiP synthesized by Abmart Biomedicine Co. Ltd. The dilution ratio of primary antibody to blocking buffer was 1:500. The antibody-antigen complex was detected with gold-labeled secondary antibody and observed using a transmission electron microscope (H-7100; Hitachi; Tokyo, Japan) running at 80 kV.

### Glutenin extraction and sodium dodecyl sulfate-polyacrylamide gel electrophoresis (SDS-PAGE)

Glutenin extraction and SDS-PAGE were performed using a Bio-Rad PROTEAN II XL electrophoresis unit based on methods previously described by [67].

### Real-time quantitative reverse transcription-polymerase chain reaction (qRT-PCR)

The PrimeScript™ RT reagent Kit with gDNA Eraser provided by TaKaRa was used for RNA purification and reverse transcription following the manufacturer’s instructions. The primers for real-time qRT-PCR were designed using Primer Premier 5.0 (Additional file 1), and ADP-ribosylation factor was selected as the internal reference gene because of its relatively stable expression levels in different tissues and samples, as reported by Paolacci et al. [68]. The transcription levels of *TaBiP* genes in three biological replicates for different treatments were quantified using qRT-PCR with a CFX96 Real-Time PCR Detection System (Bio-Rad) with SYBR-green as the intercalating dye, and the 2^-ΔΔCT^ method [69]. Real-time melting temperature curves for each of the *TaBi*P genes exhibited only a single peak, which was confirmed by agarose gel electrophoresis. The qRT-PCR efficiency was determined by serial five-fold dilutions of cDNA, and the standard curve indicated high RT-PCR efficiency rates (see Additional file 5).

## Additional files

Additional file 1:
**The primers and products of cloning of partial-length cDNA, RACE, completed cDNA, and full DNA sequences, used for real-time quantitative RT-PCR (qRT-PCR).**
Additional file 2:
**Analysis of the complete cloned** ***TaBiP*** **DNA sequences.**
Additional file 3:
**The conserved motif analysis of BiP sequences.**
Additional file 4:
**A total of 42 BiP amino acids sequences were used to construct an unrooted phylogenetic tree for analyzing the evolutionary relationships among different species.**
Additional file 5:
**qRT-PCR optimization design: double standard curves and dissolution curves of TaBiP in wheat tissue, developing seeds, and under stress.**

## Abbreviations

BiP: Binding protein
CS: Chinese spring
ERAD: ER-associated degradation
UPRE: UPR *cis* element
HSP70: 70-kDa heat shock protein
H10: Hanxuan 10
InDel: Insertion/deletion
NIL: Near-isogenic line
PDI: Protein disulfide isomerase
PBs: Protein bodies
RACE: Rapid amplification of cDNA ends
TaBiP: *Triticum aestivum* binding protein
TMD: Transmembrane domain
UPR: Unfolded proteins response
qRT-PCR: Real-time quantitative reverse transcriptional-polymerase chain reaction
