# Supplementary material for: Molecular cloning, phylogenetic analysis, and expression profiling of endoplasmic reticulum molecular chaperone BiP genes from bread wheat (Triticum aestivum L.)
Source: BMC Plant Biol. 2014 Oct 1;14:260. doi: 10.1186/s12870-014-0260-0 (PMC4189733; doi:10.1186/s12870-014-0260-0)
Supplement: Additional file 1: — The primers and products of cloning of partial-length cDNA, RACE, completed cDNA, and full DNA sequences, used for real-time quantitative RT-PCR (qRT-PCR). [file 12870_2014_260_MOESM1_ESM.pdf]

Table 1 The primers of partial-length cDNA sequence, 5’ and 3’ RACE, completed cDNA sequences and the full DNA sequences

Amplified sequences	primer name	primer sequences	PCR temperature
the partial-length cDNA sequence	BiP-F<br>BiP-R	CAATGACCAGGGTAACCGTAT<br>GTTCTCGTCCAGCCACTCC	58°C
5’UTR	5’innerTaBip1<br>5’innerTaBip2	ACTTTCTTCCAATGAGACGCTTG<br>CCATCGGTGAACCCAACCCATG	58.8°C
3’UTR	3’OuterTaBip1<br>3’innerTaBip1	CACCGGCAAGTCTGAGAAGATC<br>ATCGACCGGATGGTCAAGGA	58.8°C
completed cDNA sequences	TaBiP-F<br>TaBiP-R	GTGGCGATGGATCGGGTC<br>GTTCTCTACAACTCGGCCAACAGAC	58°C
the full DNA sequences	TaBiP-1F<br>TaBiP-1R	GTGGCGATGGATCGGGTCC<br>GTCTCGTCACCG/ACCCTCGC	58°C
	TaBiP-2F<br>TaBiP-2R	GAGGACTTTGACCACAGAA<br>AACTCGGCCAACAGACTAA	58°C

Table 2 The primers of *TaBiP*s used for real-time quantitative RT-PCR (qRT-PCR)

gene	Primer name	Primer sequences	PCR temperature
ADP	F	GCTCTCCAACAACATTGCCAAC	
	R	GCTTCTGCCTGTCACATACGC	
TaBiP1	1F	GCTATTGCCTATGGTTTGGA	58.8°C
	1R	CCTTGCCGTGCTTCTTCT	
TaBiP2	2F	GTCAAGCGTCTCATTGGAAG	58.8°C
	2R	GGTATGCCTCAGCGGTCT	
TaBiP3	3F	ATCGCTGTTTGCACTTTGTG	58.8°C
	3R	TGAGGGCGTGATACGGTTA	

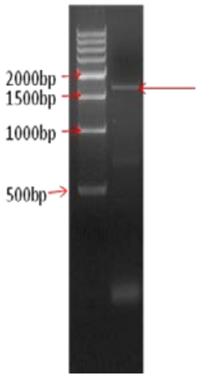

Fig. a The PCR products amplified by the specific primers BiP-F/R in the 1% agarose gel

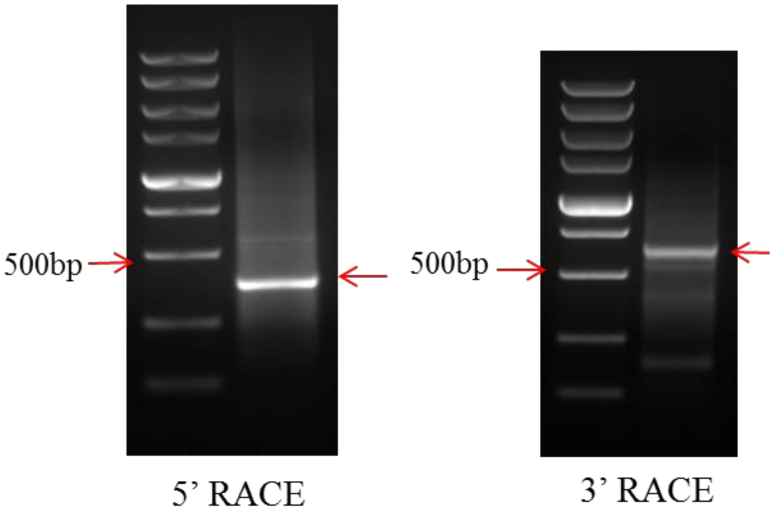

Fig. b The PCR products of 5’RACE and 3’RACE in the 1% agarose gel

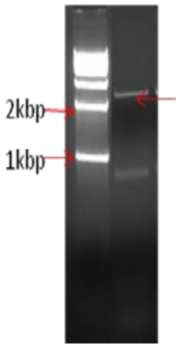

Fig. c The PCR products amplified by the specific primers TaBiP-F/R in the 1% agarose gel

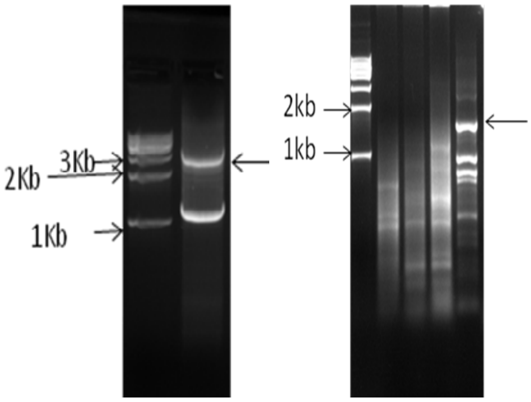

Fig. d The PCR products amplified by the specific primers BiP1F/R and BiP2F/R in the 1% agarose gel

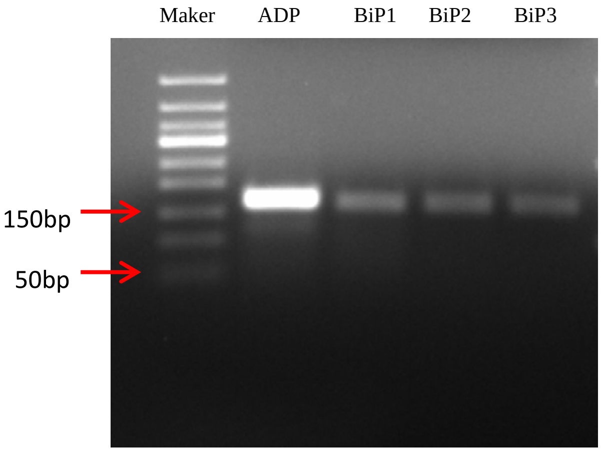

Fig. e The products of RT-PCR in the 1% agarose gel
