# Supplementary figures and images for: Molecular cloning, phylogenetic analysis, and expression profiling of endoplasmic reticulum molecular chaperone BiP genes from bread wheat (Triticum aestivum L.)
Source: BMC Plant Biol. 2014 Oct 1;14:260. doi: 10.1186/s12870-014-0260-0 (PMC4189733; doi:10.1186/s12870-014-0260-0)

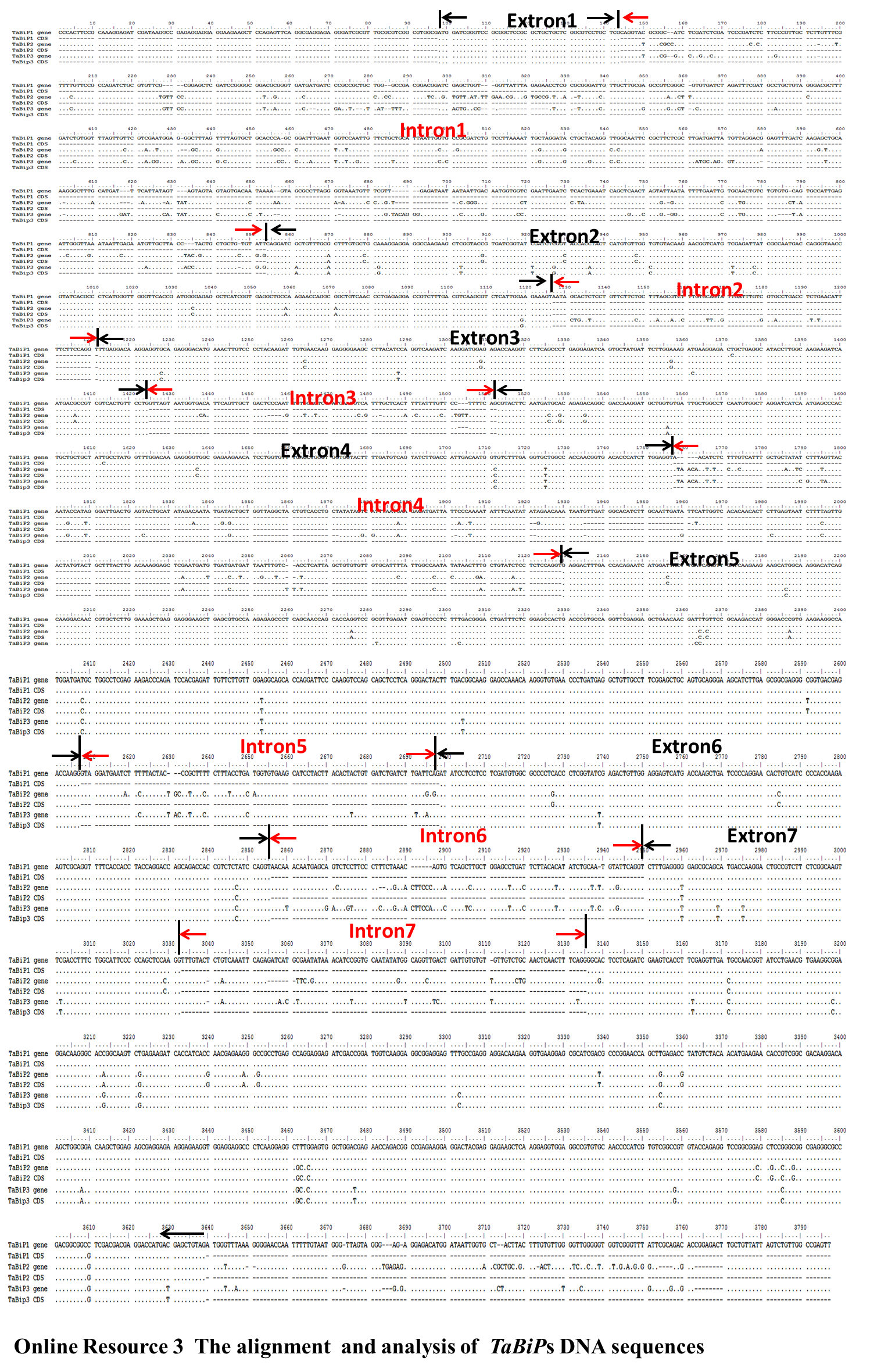

Supplement: Additional file 2: — Analysis of the complete cloned TaBiP DNA sequences. [file 12870_2014_260_MOESM2_ESM.tiff]

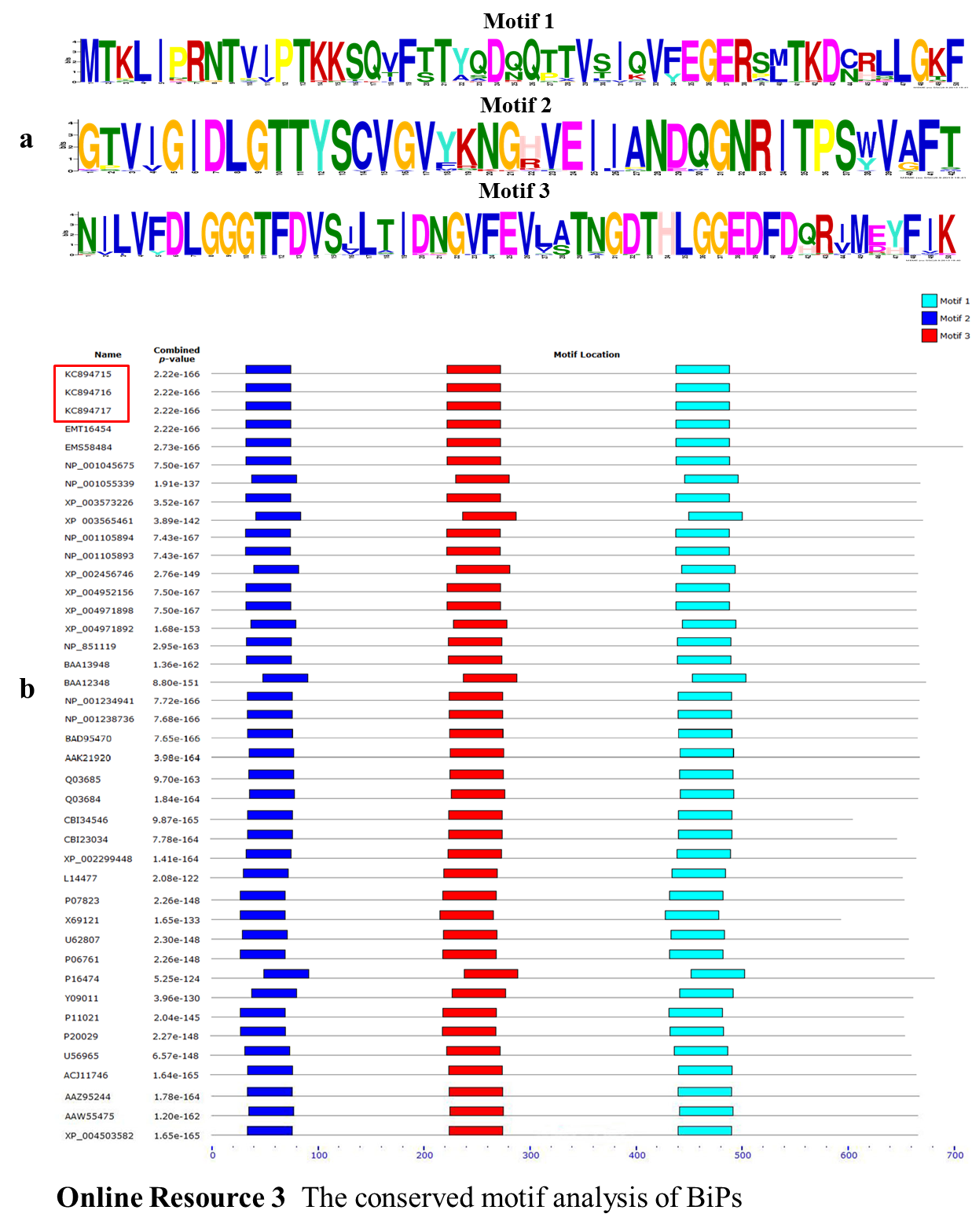

Supplement: Additional file 3: — The conserved motif analysis of BiP sequences. [file 12870_2014_260_MOESM3_ESM.tiff]
