# Supplementary material for: Molecular cloning, phylogenetic analysis, and expression profiling of endoplasmic reticulum molecular chaperone BiP genes from bread wheat (Triticum aestivum L.)
Source: BMC Plant Biol. 2014 Oct 1;14:260. doi: 10.1186/s12870-014-0260-0 (PMC4189733; doi:10.1186/s12870-014-0260-0)
Supplement: Additional file 4: — A total of 42 BiP amino acids sequences were used to construct an unrooted phylogenetic tree for analyzing the evolutionary relationships among different species. [file 12870_2014_260_MOESM4_ESM.doc]

**Additional file 4** A total of 42 BiP amino acids sequences were used to construct an unrooted phylogenetic tree for analyzing the evolutionary relationships among different species, including three from *Triticum aestivum* (*T. aestivum*), two from *Oryza sativa* (*O. sativa*), two from *Zea mays* (*Z. mays*), two from *Brachypodium distachyon* (*B. distachyon*), two from *Sorghum bicolor* (*S. bicolor*), three from *Setaria italic* (*S. italic*), one from *Aegilops tauschii* (*A. tauschii*), one from *Triticum urartu* (*T. urartr*), four from *Glycine max* (*G. max*), three from *Arabidopsis thaliana* (*A. thaliana*), two from *Nicotiana tabacum* (*N. tabacum*), and two from *Vitis vinifera* (*V. vinifera*), with the remaining sequences coming from *Plasmodium falciparum* (*P. falciparum*), *Homo sapiens* (*H. sapien*), *Mesocricetus auratus* (*M. auratus*), *Mus musculus* (*M. musculus*), *Rattus norvegicus* (*R. norvegicus*), *Xenopus laevis* (*X. laevis*) , *Trypanosoma brucei* (*T. brucei* ), *Malus pumila* (*M. pumila*), *Gossypium hirsutum* (*G. hirsutum*), *Isatis tinctoria* (*I. tinctoria*), *Neurospora crassa* (*N. crassa*), *Saccharomyces cerevisiae* (*S. cerevisiae*), *Caenorhabditis elegans* (*C. elegans*), *Populus trichocarpa* (*P. trichocarpa*)and *Cicer arietinum* (*C. arietinum*).

>*T.aestivum* BiP1 [GenBank: KC894715]

MDRVRGSALLLGVLLAGSLFALCAAKEEAKKLGTVIGIDLGTTYSCVGVYKNGHVEIIANDQGNRITPSWVGFTDGERLIGEAAKNQAAVNPERTVFDVKRLIGRKFEDKEVQRDMKLVPYKIVNKEGKPYIQVKIKDGETKVFSPEEISAMILGKMKETAEAYLGKKINDAVVTVPAYFNDAQRQATKDAGVIAGLNVARIINEPTAAAIAYGLDKRGGEKNILVFDLGGGTFDVSILTIDNGVFEVLATNGDTHLGGEDFDHRIMDYFIKLIKKKHGKDISKDNRALGKLRREAERAKRALSNQHQVRVEIESLFDGTDFSEPLTRARFEELNNDLFRKTMGPVKKAMDDAGLEKTQIHEIVLVGGSTRIPKVQQLLRDYFDGKEPNKGVNPDEAVAFGAAVQGSILSGEGGDETKDILLLDVAPLTLGIETVGGVMTKLIPRNTVIPTKKSQVFTTYQDQQTTVSIQVFEGERSMTKDCRLLGKFDLSGIPPAPRGTPQIEVTFEVDANGILNVKAEDKGTGKSEKITITNEKGRLSQEEIDRMVKEAEEFAEEDKKVKERIDARNQLETYVYNMKNTVGDKDKLADKLESEEKEKVEEALKEALEWLDENQTAEKEDYEEKLKEVEAVCNPIVSAVYQRSGGAPGGEGADGGVDDEDHDEL

>*T.aestivum* BiP2 [GenBank: KC894716]

MDRVRGSALLLGVLLAGSLFALCAAKEEAKKLGTVIGIDLGTTYSCVGVYKNGHVEIIANDQGNRITPSWVGFTDGERLIGEAAKNQAAVNPERTVFDVKRLIGRKFEDKEVQRDMKLVPYKIVNKEGKPYIQVKIKDGETKVFSPEEISAMILGKMKETAEAYLGKKINDTVVTVPAYFNDAQRQATKDAGVIAGLNVARIINEPTAAAIAYGLDKRGGEKNILVFDLGGGTFDVSILTIDNGVFEVLATNGDTHLGGEDFDHRIMDYFIKLIKKKHGKDISKDNRALGKLRREAERAKRALSNQHQVRVEIESLFDGTDFSEPLTRARFEELNNDLFRKTMGPVKKAMDDAGLEKTQIHEIVLVGGSTRIPKVQQLLRDYFDGKEPNKGVNPDEAVAFGAAVQGSILSGEGGDETKDILLLDVAPLTLGIETVGGVMTKLIPRNTVIPTKKSQVFTTYQDQQTTVSIQVFEGERSMTKDCRLLGKFDLSGIPPAPRGTPQIEVTFEVDANGILNVKAEDKGTGKSEKITITNEKGRLSQEEIDRMVKEAEEFAEEDKKVKERIDARNQLETYVYNMKNTVGDKDKLADKLESEEKEKVEEALKEALEWLDENQTAEKEDYEEKLKEVEAVCNPIVSAVYQRSGGAPGGEGADGGVDDEDHDEL

>*T.aestivum* BiP3 [GenBank: KC894717]

MDRVRGSALLLGVLLAGSLFALCAAKEEAKKLGTVIGIDLGTTYSCVGVYKNGHVEIIANDQGNRITPSWVGFTDGERLIGEAAKNQAAVNPERTVFDVKRLIGRKFEDKEVQRDMKLVPYKIVNKEGKPYIQVKIKDGETKVFSPEEISAMILGKMKETAEAYLGKKINDAVVTVPAYFNDAQRQATKDAGVIAGLNVARIINEPTAAAIAYGLDKRGGEKNILVFDLGGGTFDVSILTIDNGVFEVLATNGDTHLGGEDFDHRIMDYFIKLIKKKHGKDISKDNRALGKLRREAERAKRALSNQHQVRVEIESLFDGTDFSEPLTRARFEELNNDLFRKTMGPVKKAMDDAGLEKTQIHEIVLVGGSTRIPKVQQLLRDYFDGKEPNKGVNPDEAVAFGAAVQGSILSGEGGDETKDILLLDVAPLTLGIETVGGVMTKLIPRNTVIPTKKSQVFTTYQDQQTTVSIQVFEGERSMTKDCRLLGKFDLSGIPPAPRGTPQIEVTFEVDANGILNVKAEDKGTGKSEKITITNEKGRLSQEEIDRMVKEAEEFAEEDKKVKERIDARNQLETYVYNMKNTVGDKDKLADKLESEEKEKVEEALKEALEWLDENQTAEKEDYEEKLKEVEAVCNPIVSAVYQRSGGAPGGEGADGGVDDEDHDEL

>*O.sativa* BiP1 [GenBank: NP_001045675]

MDRVRGCAFLLGVLLAGSLFAFSVAKEETKKLGTVIGIDLGTTYSCVGVYKNGHVEIIANDQGNRITPSWVAFTDSERLIGEAAKNQAAVNPERTIFDVKRLIGRKFEDKEVQRDMKLVPYKIVNKDGKPYIQVKIKDGENKVFSPEEVSAMILGKMKETAEAYLGKKINDAVVTVPAYFNDAQRQATKDAGVIAGLNVARIINEPTAAAIAYGLDKKGGEKNILVFDLGGGTFDVSILTIDNGVFEVLATNGDTHLGGEDFDQRIMEYFIKLIKKKYSKDISKDNRALGKLRREAERAKRALSNQHQVRVEIESLFDGTDFSEPLTRARFEELNNDLFRKTMGPVKKAMDDAGLEKSQIHEIVLVGGSTRIPKVQQLLRDYFEGKEPNKGVNPDEAVAYGAAVQGSILSGEGGDETKDILLLDVAPLTLGIETVGGVMTKLIPRNTVIPTKKSQVFTTYQDQQTTVSIQVFEGERSMTKDCRLLGKFDLSGIPAAPRGTPQIEVTFEVDANGILNVKAEDKGTGKSEKITITNEKGRLSQEEIDRMVREAEEFAEEDKKVKERIDARNQLETYVYNMKNTVGDKDKLADKLESEEKEKVEEALKEALEWLDENQTAEKEEYEEKLKEVEAVCNPIISAVYQRTGGAPGGGADGEGGVDDEHDEL

>*O.sativa* BiP2 [GenBank: NP_001055339]

MARGATWTRRLHLHGLFLAVLLLLTLPAGSTAAAGGGGGTVIGIDLGTTYSCVGVYRNGHVEIIANDQGNRITPSWVAFTGGGERLIGEAAKNQAAANPGRTVYDAKRLIGRRFADAEVQRDMRLLPFAVVDKGGKPHVRVEVRGGDVRLLSPEEVSAMVLARMKETAEAYLGEEVTRAVVTVPAYFNDAQRQATKDAATIAGLAVERILNEPTAAALAYGVGKEGAGGKNVLVFDLGGGTFDVSVLAIDGGVYEVLATNGDTHLGGEDFDQRVMEHFVELVRRKHGRDIAGDARALGKLRRECERAKRALSIQHQVRVEVESLFDGVDLSEPLSRARFEELNNDLFRKTMAPVRKAMADARLSNADIDEIVLVGGSTRIPKVRQLLRDYFGGKQPNQGVNPDEAVAYGAAIQANIVGGDTDNKTRDMVVLDVTPLTLGLETAGGVMATLIPRNTPVPTKRAQLFSTYKDKQTTVTVKVFEGERSMTRDNRLLGRFDLAGIAPAPRGAPQIEVAFEVDADGILSVSAADRATGRSERITISGDDRKTSREEIDRMLGEAEEFADEDRRHRERAGARNSLEAYVYGVKNAVVGGEMAGAMDGGEKEKVEAAVMEAYEWLDGNQDVGKEEYEEKLRELEDVCNPVMSAVYQRSGGSRRDGDGGGDDDHDEL

>*Z.mays* BiP1 [GenBank: NP_001105894]

MDRVRGSAFLLGVLLAGSLFAFSVAKEETKKLGTVIGIDLGTTYSCVGVYKNGHVEIIANDQGNRITPSWVAFTDSERLIGEAAKNQAAVNPERTIFDVKRLIGRKFQDKEVQRDMKLVPYKIINKDGKPYIQVKIKDGENKVFSPEEISAMILGKMKDTAEAYLGKKINDAVVTVPAYFNDAQRQATKDAGVIAGLNVARIINEPTAAAIAYGLDKKGGEKNILVFDLGGGTFDVSILTIDNGVFEVLATNGDTHLGGEDFDQRIMEYFIKLIKKKYSKDIGKDNRALGKLRREAERAKRALSNQHQVRVEIESLFDGTDFSEPLTRARFEELNNDLFRKTMGPVKKAMEDAGLEKSQIHEIVLVGGSTRIPKVQQLLKDYFNGKEPNKGVNPDEAVAFGAAVQGSILSGEGGDETKDILLLDVAPLTLGIETVGGVMTKLIPRNTVIPTKKSQVFTTYQDQQTTVSIQVFEGERSMTKDCRLLGKFDLNGIPSAPRGTPQIEVTFEVDANGILNVKAEDKGTGKSEKITITNEKGRLSQEEIDRMVREAEEFAEEDKKVKERIDARNQLETYVYNMKNTVGDKDKLADKLEAEEKEKVEEALKEALEWLDDNQSAEKEDYEEKLKEVEAVCNPIVSAVYQRSGGAPGGDADGGVDDDHDEL

>*Z.mays* BiP2 [GenBank: NP_001105893]

MDRARGSAFLLGVLLAGSLFAFSVAKEETKKLGTVIGIDLGTTYSCVGVYKNGHVEIIANDQGNRITPSWVAFTDSERLIGEAAKNQAAVNPERTIFDVKRLIGRKFADKEVQRDMKLVPYKIINKDGKPYIQVKIKDGENKVFSPEEISAMILGKMKDTAEAYLGKKINDAVVTVPAYFNDAQRQATKDAGVIAGLNVARIINEPTAAAIAYGLDKKGGEKNILVFDLGGGTFDVSILTIDNGVFEVLATNGDTHLGGEDFDQRIMEYFIKLIKKKYSKDISKDNRALGKLRREAERAKRALSNQHQVRVEIESLFDGTDFSEPLTRARFEELNNDLFRKTMGPVKKAMEDAGLEKSQIHEIVLVGGSTRIPKVQQLLRDYFDGKEPNKGVNPDEAVAFGAAVQGSILSGEGGDETKDILLLDVAPLTLGIETVGGVMTKLIPRNTVIPTKKSQVFTTYQDQQTTVSIQVFEGERSMTKDCRLLGKFDLNGIAPAPRGTPQIEVTFEVDANGILNVKAEDKGTGKSEKITITNEKGRLSQEEIDRMVREAEEFAEEDKKVKERIDARNQLETYVYNMKNTVGDKDKLADKLEAEEKEKVEEALKEALEWLDDNQSAEKEDYEEKLKEVEAVCNPIVSAVYQRSGGAPGGDADGGVDDDHDEL

>*B.distachyon* BiP1 [GenBank: XP_003573226]

MDRVRGSLLLLGVLLAGSLFAFSAAKEEAKKLGTVIGIDLGTTYSCVGVYKNGHVEIIANDQGNRITPSWVGFTDSERLIGEAAKNQAAVNPERTIFDVKRLIGRKFEDKEVQRDMKLVPYKIVNRDGKPYIQVKIKDGENKVFSPEEISAMILGKMKETAEAYLGKKINDAVVTVPAYFNDAQRQATKDAGVIAGLNVARIINEPTAAAIAYGLDKKGGEKNILVFDLGGGTFDVSILTIDNGVFEVLATNGDTHLGGEDFDHRIMEYFIKLIKKKYSKDISKDNRALGKLRREAERAKRALSNQHQVRVEIESLFDGTDFSEPLTRARFEELNNDLFRKTMGPVKKAMDDAGLEKSQIHEIVLVGGSTRIPKVQQLLRDYFEGKEPNKGVNPDEAVAFGAAVQGSILSGEGGDETKDILLLDVAPLTLGIETVGGVMTKLIPRNTVIPTKKSQVFTTYQDQQTTVSIQVFEGERSMTKDCRLLGKFDLSGIPPAPRGTPQIEVTFEVDANGILNVKAEDKGTGKSEKITITNEKGRLSQEEIDRMVKEAEEFAEEDKKVKERIDARNQLETYVYNMKNTVGDKDKLADKLESEEKEKVEEALKEALEWLDENQSAEKEDYEEKLKEVEAVCNPIVSAVYQRSGGAPGGEDGAGGVDDEEHDEL

>*B.distachyon* BiP2 [GenBank: XP_003565461]

MARGTSTTALLLGLFLVGFLVAPSAAAADSKGDQQPANKASGGPVIGIDLGTTYSCVGVYRNGHVEIIANDQGNRITPSWVAFTDSGERLIGEAAKNQAASNPLRTIYDAKRLIGRQYGDAEVHKDMKHLPYKIVEKRGKPHMEVEVKDGDVRTLSPEEVSAMVLTRMKETAEAFLGEPVKDAVITIPAYFNDAQRQATKDAGAIAGLNVVRLINEPTAAAIAYGLDNKAKDAKEERNVLVFDLGGGTFDVSVLTIDNGVFEVLATNGDTHLGGEDFDHRLMDYLVKLVKRKHGKDVSHDARALGKLRRECERAKRALSSQHQVRVEIESLFDGVDLSEPLTRARFEELNSDLFRKTMTPVKKAMADAGLAKGDIHEVVLVGGSTRIPKIQQFLKDYFDGKEPSKGVNPDEAVAYGAAVQGSIVRGDNAEKLVVLDVTPLTLGIETAGGVMTPLIPRGTVIPTRKTKTFTTYQDRQTTVSVVVFEGERSMTKDNKQLGKFDLTGIAPAPRGTPQIEVTFEVDVNGILHVKAADKGTGKSEKIQITSAADRRITQEEIDRMVREAEEFAEEDRKVRERVDARNRMEAYVYHVRTTVDGEAGQGMDGGDKERVREAAREASEWIDENPEADKDEYVEKLKELEDLCNPVFAAADSHKSGGGHDEAEEDDHDEL

>*S.bicolor* BiP1 [GenBank: XP_002456746]

MAARGGWTKLLFALLVAGLLPVVAPTRTYAAAAAEGGGARGTVIGIDLGTTYSCVGVYRNGRVEIIANDQGNRITPSWVAFTDGGERLIGEAAKNQAAANPERTIYDAKRLIGRQFDDAVVQRDMKLLSYDVVERNGKPHVRVQVREGDVRELSPEEVSAMVLTKMKETAEAYLGEKVTDAVVTVPAYFDDAQRQATKDAGTIAGLNVRRIINEPTAAAIAYGIDDKGPEKKVLVFDLGGGTFDVSILAIDNGVFEVLATNGDTHLGGEDFDQRVMDYFIKLIKRRHGRDISGDARALGKLRRECERAKRALSNQHQVRVEIEALFDGVDISEQLTRARFEELNSDLFRKTMVPVKKAMADARLQKSDIDEIVLVGGSTRIPKVQQLLKDYFNGKEPSRGINPDEAVAYGAAVQGGILSGHGESMLVIDVTPLTLGIETVGGVMTKLIPRNTVIPTKKTQVFTTYQDRQTTVSIMVFQGERSMTKDNELLGKFDLSGIPPAPKGTPQIEVTFEVDVNGILHVKAADKGTGRSEKVTITSDARRVSQEEMDRMVHEAEEFAEEDRKARERVDARNKLETYVYQVKSAVVDDSNMADKMNADEKEKVEEAVREANEWIEVNSDADKEDYEEKLKELEDVCSPVISAVYQRSGGGAPADDTYDEDDHDEL

>*S.bicolor*BiP 2 [GenBank: XP_004952156]

MDRVRGSAFLLGVLLAGSLFAFSVAKEETKKLGTVIGIDLGTTYSCVGVYKNGHVEIIANDQGNRITPSWVAFTDSERLIGEAAKNQAAVNPERTIFDVKRLIGRKFEDKEVQRDMKLVPYKIINKDGKPYIQVKIKDGENKVFSPEEISAMILGKMKDTAEAYLGKKINDAVVTVPAYFNDAQRQATKDAGVIAGLNVARIINEPTAAAIAYGLDKKGGEKNILVFDLGGGTFDVSILTIDNGVFEVLATNGDTHLGGEDFDQRIMEYFIKLIKKKYSKDISKDNRALGKLRREAERAKRALSNQHQVRVEIESLFDGTDFSEPLTRARFEELNNDLFRKTMGPVKKAMEDAGLQKSQIHEIVLVGGSTRIPKVQQLLRDYFDGKEPNKGVNPDEAVAFGAAVQGSILSGEGGDETKDILLLDVAPLTLGIETVGGVMTKLIPRNTVIPTKKSQVFTTYQDQQTTVSIQVFEGERSMTKDCRLLGKFDLSGIPPAPRGTPQIEVTFEVDANGILNVKAEDKGTGKSEKITITNEKGRLSQEEIDRMVKEAEEFAEEDKKVKERIDARNQLETYVYNMKNTVGDKDKLADKLEAEEKEKVEEALKEALEWLDDNQSAEKDEYVEKLKEVEAVCNPIVSAVYQRSGGAPGGGDDSEGGVDDDHDEL

>*S.italica* BiP1 [GenBank: XP_004971898]

MDRVRGSAFLLGVLLAGSLFAFSVAKEETKKLGTVIGIDLGTTYSCVGVYKNGHVEIIANDQGNRITPSWVAFTDSERLIGEAAKNQAAVNPERTIFDVKRLIGRKFQDKEVQRDMKLVPYNIVNKEGKPYIQVKIKDGENKVFSPEEISAMILGKMKDTAEAYLGKKINDAVVTVPAYFNDAQRQATKDAGVIAGLNVARIINEPTAAAIAYGLDKKGGEKNILVFDLGGGTFDVSILTIDNGVFEVLATNGDTHLGGEDFDQRIMEYFIKLIKKKYSKDISKDNRALGKLRREAERAKRALSNQHQVRVEIESLFDGTDFSEPLTRARFEELNNDLFRKTMGPVKKAMEDAGLEKSQIHEIVLVGGSTRIPKVQQLLRDYFDGKEPSKGVNPDEAVAYGAAVQGSILSGEGGDETKDILLLDVAPLTLGIETVGGVMTKLIPRNTVIPTKKSQVFTTYQDQQTTVSIQVFEGERSMTKDCRLLGKFDLSGIPPAPRGTPQIEVTFEVDANGILNVKAEDKGTGKSEKITITNEKGRLSQEEIDRMVREAEEFAEEDKKVKEKIDARNQLETYVYNMKNTIGDKDKLADKLESEEKEKVEEALKEALEWLDDNQSAEKEDYEEKLKEVEAVCNPIISAVYQKSGGAPGGGADEEGGVDD

DHDEL

>*S.italica* BiP2 [GenBank: XP_004971892]

MAPGRVWTLLFALLLANLVAPIHAAAPSHGGGGDKNTGTVIGIDLGTTYSCVGVYRNGHVEIIANDQGNRITPSWVAFTDGGERLIGEAAKNQAAANPERTIYDAKRLIGRQFDDAVVQRDMKLLPYAVVDRNGKPHMRVQVRDGDVREFSPEEVSAMVLTKMKETAEAYLGEKVTHAVVTVPAYFNDAQRQATKDAGVIAGLTVLRIVNEPTAAAIAYGIDKKGAEKNVLVFDLGGGTFDVSVLAIDNGVFEVLATNGDTHLGGEDFDQRVMDYFIKLIKRKHGRDIAGDARALGKLRRECERAKRTLSNQHQVRVEIEALFDGVDFSEQLTRARFEELNNDLFRKTMVPVKKAMADAGLQKGDIDEIVLVGGSTRIPKVQQLLKDYFNGKEPNKGVNPDEAVAYGAAVQGSILSGHVDEKTQGMILLDVAPLTLGMETVGGVMTKLITRNTVVPTKKTQVFTTYQDRQTTVSIKVFEGERSMTKDNRLLGKFDLAGIPPAPRGTPQIEVTFEVDANGILHVQAADKGTGKSEKITITSDDRRLSQEEIDRMVREAEEFAEEDKKVRERVDARNKLETYIYQVKSAVDDTKMTDKMDVEDKEKMEEAVREANEWLEGNSLDADKEDYEEKLKELEDVCNPIISAVYQKSSGSPGEDNYDEDDHDEL

>*S.italica* BiP3 [GenBank: XP_004952156]

MAPGRVWTLLFALLVADLVAPIHAAAPPHGGGGDKNTGTVIGIDLGTTYSCVGVYRNGHVEIIANDQGNRITPSWVAFTDGGERLIGEAAKNQAAANPERTIYDAKRLIGRQFDDAVVQRDMKLLPYAVVDRNGKPHVRVQVRDGDVREFSPEEVSAMVLTKMKETAEAYLGEKVTHAVVTVPAYFNDAQRQATKDAGVIAGLTVLRIVNEPTAAAIAYGIDKKGAEKNVLVFDLGGGTFDVSVLAIDNGVFEVLATNGDTHLGGEDFDQRVMDYFIKLIKRKHARDIAGDARALGKLRRECERAKRALSNQHQVRVEIEALFDGVDFSEQLTRARFEELNNDLFRKTMVPVKKAMADAGLNKGDIDEIVLVGGSTRIPKVQQLLKDYFNGKEPSKGVNPDEAVAYGAAVQGSILSGHVDENTKGMILLDVAPLTLGMETAGGVMTKLITRNTVVPTKKTQVFTTYQDRQTTVSIKVFEGERSMTKDNRLLGKFDLTGIPPAPRGTPQIEVTFEVDANGILHVTAADKGTGKSEKITITSDDRRLSQEEVDRMVREAEEFAEEDRKVRERVDARNKLETYVYQVKSTMDDTKMADKMDVEDKERMEEAVREANEWLEGNSLDADKEDYEDKLKQLEDVCNPIISAVYQKSSGSPGEDNYDEDDHDEL

>*A.tauschii* [GenBank: EMT16454]

MDRVRGSALLLGVLLAGSLFALCAAKEEAKKLGTVIGIDLGTTYSCVGVYKNGHVEIIANDQGNRITPSWVGFTDGERLIGEAAKNQAAVNPERTVFDVKRLIGRKFEDKEVQRDMKLVPYKIVNKEGKPYIQVKIKDGETKVFSPEEISAMILGKMKETAEAYLGKKINDAVVTVPAYFNDAQRQATKDAGVIAGLNVARIINEPTAAAIAYGLDKRGGEKNILVFDLGGGTFDVSILTIDNGVFEVLATNGDTHLGGEDFDHRIMDYFIKLIKKKHGKDISKDNRALGKLRREAERAKRALSNQHQVRVEIESLFDGTDFSEPLTRARFEELNNDLFRKTMGPVKKAMDDAGLEKTQIHEIVLVGGSTRIPKVQQLLRDYFDGKEPNKGVNPDEAVAFGAAVQGSILSGEGGDETKDILLLDVAPLTLGIETVGGVMTKLIPRNTVIPTKKSQVFTTYQDQQTTVSIQVFEGERSMTKDCRLLGKFDLSGIPPAPRGTPQIEVTFEVDANGILNVKAEDKGTGKSEKITITNEKGRLSQEEIDRMVKEAEEFAEEDKKVKERIDARNQLETYVYNMKNTVGDKDKLADKLESEEKEKVEEALKEALEWLDENQTAEKEDYEEKLKEVEAVCNPIVSAVYQRSGGAPGGEGADGGVDDEDHDEL

>*T.urartu* [GenBank: EMS58484]

MDRVRGSALLLGVLLAGSLFALCAAKEEAKKLGTVIGIDLGTTYSCVGVYKNGHVEIIANDQGNRITPSWVGFTDGERLIGEAAKNQAAVNPERTVFDVKRLIGRKFEDKEVQRDMKLVPYKIVNKEGKPYIQVKIKDGETKVFSPEEISAMILGKMKETAEAYLGKKINDAVVTVPAYFNDAQRQATKDAGVIAGLNVARIINEPTAAAIAYGLDKRGGEKNILVFDLGGGTFDVSILTIDNGVFEVLATNGDTHLGGEDFDHRIMDYFIKLIKKKHGKDISKDNRALGKLRREAERAKRALSNQHQVRVEIESLFDGTDFSEPLTRARFEELNNDLFRKTMGPVKKAMDDAGLEKTQIHEIVLVGGSTRIPKVQQLLRDYFDGKEPNKGVNPDEAVAFGAAVQGSILSGEGGDETKDILLLDVAPLTLGIETVGGVMTKLIPRNTVIPTKKSQVFTTYQDQQTTVSIQVFEGERSMTKDCRLLGKFDLSGIPPAPRGTPQIEVTFEVDANGILNVKAEDKGTGKSEKITITNEKGRLSQEEIDRMVKEAEEFAEEDKKVKERIDARNQLETYVYNMKNTVGDKDKLADKLESEEKEKVEEALKEALEWLDENQTAEKEDYEEKLKEVEAVCNPIVSAGYQRSGGAPGGGGADGGGGEAGPDRREMEKLKEVEAVCNPIVSAVYQRSGGAPGGEGADGGVDDEDHDEL

>*G.max* BiP A [GenBank: NP_001234941]

MAGSWARRSLIVLAIISFGCLFAISIAKEEATKLGTVIGIDLGTTYSCVGVYKNGHVEIIANDQGNRITPSWVAFTDSERLIGEAAKNLAAVNPERTIFDVKRLIGRKFEDKEVQRDMKLVPYKIVNKDGKPYIQVKIKDGETKVFSPEEISAMILTKMKETAEAFLGKKINDAVVTVPAYFNDAQRQATKDAGVIAGLNVARIINEPTAAAIAYGLDKKGGEKNILVFDLGGGTFDVSILTIDNGVFEVLATNGDTHLGGEDFDQRIMEYFIKLIKKKHGKDISKDNRALGKLRREAERAKRALSSQHQVRVEIESLFDGVDFSEPLTRARFEELNNDLFRKTMGPVKKAMEDAGLQKSQIDEIVLVGGSTRIPKVQQLLKDYFDGKEPNKGVNPDEAVAYGAAVQGSILSGEGGEETKDILLLDVAPLTLGIETVGGVMTKLIPRNTVIPTKKSQVFTTYQDQQTTVSIQVFEGERSLTKDCRLLGKFDLSGIPPAPRGTPQIEVTFEVDANGILNVKAEDKGGKSEKITITNEKGRLSQEEIERMVREAEEFAEEDKKVKERIDARNSLETYVYNMKNQISDKDKLADKLESDEKEKIETAVKEALEWLDDNQSMEKEDYEEKLKEVEAVCNPIISAVYQRSGGAPGGGGASGEEDEDDSHDEL

>*G.max* BiPB [GenBank: NP_001238736]

MAGSWARRSLIVLAIISFGCLFAISIAKEEATKLGTVIGIDLGTTYSCVGVYKNGHVEIIANDQGNRITPSWVAFTDSERLIGEAAKNQAAVNPERTIFDVKRLIGRKFEDKEVQKDMKLVPYKIVNKDGKPYIQVKIKDGETKVFSPEEISAMVLIKMKETAEAFLGKKINDAVVTVPAYFNDAQRQATKDAGVIAGLNVARIINEPTAAAIAYGLDKKGGEKNILVFDLGGGTFDVSILTIDNGVFEVLATNGDTHLGGEDFDQRIMEYFIKLIKKKHGKDISKDNRALGKLRREAERAKRALSSQHQVRVEIESLFDGVDFSEPLTRARFEELNNDLFRKTMGPVKKAMEDAGLQKSQIDEIVLVGGSTRIPKVQQLLKDYFDGKEPNKGVNPDEAVAYGAAVQGSILSGEGGEETKDILLLDVAPLTLGIETVGGVMTKLIPRNTVIPTKKSQVFTTYQDQQTTVSIQVFEGERSLTKDCRLLGKFDLSGIPPAPRGTPQIEVTFEVDANGILNVKAEDKGTGKSEKITITNEKGRLSQEEIDRMVREAEEFAEEDKKVKERIDARNSLETYVYNMKNQVSDKDKLADKLESDEKEKIETAVKEALEWLDDNQSVEKEDYEEKLKEVEAVCNPIISAVYQRSGGAPGGAGGEGEDEDDSHDEL

>*G.max* BiPC [GenBank: BAD95470]

MARSFSRGSLLPLAIVSLVCLFVISIAKEEATKLGTVIGIDLGTTYSCVGVYKNGHVEIIANDQGNRITPSWVAFTDSERLIGEAAKNLAAVNPERTIFDVKRLIGRKFEDKEVQRDMKLVPYKIVNKDGKPYIQVKIKDGETKVFSPEEISAMILTKMKETAEAFLGKKINDAVVTVPAYFNDAQRQATKDAGVIAGLNVARIINEPTAAAIAYGLDKKGGEKNILVFDLGGGTFDVSILTIDNGVFEVLATNGDTHLGGEDFDQRIMEYFIKLIKKKHGKDISKDSRALGKLRREAERAKRALSSQHQVRVEIESLFDGVDFSEPLTRARFEELNNDLFRKTMGPVKKAMEDAGLQKSQIDEIVLVGGSTRIPKVQQLLKDYFDGKEPNKGVNPDEAVAYGAAVQGSILSGEGGEETKDILLLDVAPLTLGIETVGGVMTKLIPRNTVIPTKKSQVFTTYQDQQTTVSIQVFEGERSLTKDCRLLGKFELSGIPPAPRGTPQIEVTFEVDANGILNVKAEDKGTGKSEKITITNEKGRLSQEEIERMVREAEEFAEEDKKVKERIDARNSLETYVYNMKNQIGDKDKLADKLESDEKEKIETAVKEALEWLDDNQSVEKEEYEEKLKEVEAVCNPIISAVYQRSGGAPGGGASGEEDDDSHDEL

>*G.max* BiPD [GenBank: AAK21920]

MACSFSRGSLLPLAIIVSLGCLFAISIAKEEATKLGTVIGIDLGTTYSCVGVYKNGHVEIIANDQGNRITPSWVAFTDSERLIGEAAKNLAAVNPERVIFDVKRLIGRKFEDKEVQRDMKLVPYKIVNKDGKPYIQVKIKDGETKVFSPEEISAMILTKMKETAEAFLGKKINDAVVTVPAYFNDAQRQATKDAGVIAGLNVARIINEPTAAAIAYGLDKKGGEKNILVFDLGGGTFDVSILTIDNGVFEVLATNGDTHLGGEDFDQRIMEYFIKLINKKHKKDISKDSRALGKLRREAERAKRALSSQHQVRVEIESLFDGVDFSEPLTRARFEELNNDLFRKTMGPVKKAMEDAGLQKNQIDEIVLVGGSTRIPKVQQLLKDYFDGKEPNKGVNPDEAVAYGAAVQGSILSGEGGEETKDILLLDVAPLTLGIETVGGVMTKLIPRNTVIPTKKSQVFTTYQDQQSTVSIQVFEGERSLTKDCRLLGKFELSGIPPAPRGTPQIEVTFEVDANGILNVKAEDKGTGKSEKITITNEKGRLSQEEIERMVREAEEFAEEDKKVKERIDARNSLETYVYNMKNQVSDKDKLADKLESDEKEKIETAVKEALEWLDDNQSVEKEEYEEKLKEVEAVCNPIISAVYQRSGGAPGGGASGEDDDEDSHDEL

>*A.thaliana* BiP1 [GenBank: NP_861119]

MARSFGANSTVVLAIIFFGCLFAFSTAKEEATKLGSVIGIDLGTTYSCVGVYKNGHVEIIANDQGNRITPSWVGFTDSERLIGEAAKNQAAVNPERTVFDVKRLIGRKFEDKEVQKDRKLVPYQIVNKDGKPYIQVKIKDGETKVFSPEEISAMILTKMKETAEAYLGKKIKDAVVTVPAYFNDAQRQATKDAGVIAGLNVARIINEPTAAAIAYGLDKKGGEKNILVFDLGGGTFDVSVLTIDNGVFEVLSTNGDTHLGGEDFDHRIMEYFIKLIKKKHQKDISKDNKALGKLRRECERAKRALSSQHQVRVEIESLFDGVDLSEPLTRARFEELNNDLFRKTMGPVKKAMDDAGLQKSQIDEIVLVGGSTRIPKVQQLLKDFFEGKEPNKGVNPDEAVAYGAAVQGGILSGEGGDETKDILLLDVAPLTLGIETVGGVMTKLIPRNTVIPTKKSQVFTTYQDQQTTVSIQVFEGERSLTKDCRLLGKFDLTGVPPAPRGTPQIEVTFEVDANGILNVKAEDKASGKSEKITITNEKGRLSQEEIDRMVKEAEEFAEEDKKVKEKIDARNALETYVYNMKNQVSDKDKLADKLEGDEKEKIEAATKEALEWLDENQNSEKEEYDEKLKEVEAVCNPIITAVYQRSGGAPGAGGESSTEEEDESHDEL

>*A.thaliana* BiP2 [GenBank: BAA13948]

MARSFGANSTVVLAIIFFGCLFALSSAIEEATKLGSVIGIDLGTTYSCVGVYKNGHVEIIANDQGNRITPSWVGFTDSERLIGEAAKNQAAVNPERTVFDVKRLIGRKFEDKEVQKDRKLVPYQIVNKDGKPYIQVKIKDGETKVFSPEEISAMILTKMKETAEAYLGKKIKDAVVTVPA

YFNDAQRQATKDAGVIAGLNVARIINEPTAAAIAYGLDKKGGEKNILVFDLGGGTFDVSVLTIDNGVFEVLSTNGDTHLGGEDFDHRVMEYFIKLIKKKHQKDISKDNKALGKLRRECERAKRALSSQHQVRVEIESLFDGVDFSEPLTRARFEELNNDLFRKTMGPVKKAMDDAGLQKSQIDEIVLVGGSTRIPKVQQLLKDFFEGKEPNKGVNPDEAVAYGAAVQGGILSGEGGDETKDILLLDVAPLTLGIETVGGVMTKLIPRNTVIPTKKSQVFTTYQDQQTTVSIQVFEGERSLTKDCRLLGKFDLNGIPPAPRGTPQIEVTFEVDANGILNVKAEDKASGKSEKITITNEKGRLSQEEIDRMVKEAEEFAEEDKKVKEKIDARNALETYVYNMKNQVNDKDKLADKLEGDEKEKIEAATKEALEWLDENQNSEKEEYDEKLKEVEAVCNPIITAVYQRSGGAPGGAGGESSTEEEDESHDEL

>*A.thaliana* BiP3 [GenBank: BAA12348]

MIFIKENTAKMTRNKAIACLVFLTVLDFLMNIGAALMSSLAIEGEEQKLGTVIGIDLGTTYSCVGVYHNKHVEIIANDQGNRITPSWVAFTDTERLIGEAAKNQAAKNPERTIFDPKRLIGRKFDDPDVQRDIKFLPYKVVNKDGKPYIQVKVKGEEKLFSPEEISAMILTKMKETAEAFLGKKIKDAVITVPAYFNDAQRQATKDAGAIAGLNVVRIINEPTGAAIAYGLDKKGGESNILVYDLGGGTFDVSILTIDNGVFEVLSTSGDTHLGGEDFDHRVMDYFIKLVKKKYNKDISKDHKALGKLRRECELAKRSLSNQHQVRVEIESLFDGVDFSEPLTRARFEELNMDLFKKTMEPVKKALKDAGLKKSDIDEIVLVGGSTRIPKVQQMLKDFFDGKEPSKGTNPDEAVAYGAAVQGGVLSGEGGEETQNILLLDVAPLSLGIETVGGVMTNIIPRNTVIPTKKSQVFTTYQDQQTTVTINVYEGERSMTKDNRELGKFDLTGILPAPRGVPQIEVTFEVDANGILQVKAEDKVAKTSQSITITNDKGRLTEEEIEEMIREAEEFAEEDKIMKEKIDARNKLETYVYNMKSTVADKEKLAKKISDEDKEKMEGVLKEALEWLEENVNAEKEDYDEKLKEVELVCDPVIKSVYEKTEGENEDDDGDDHDEL

>*N.tabacum* BiP4 [SWISS-PORT: Q03685]

MAGAWKRRASLIVFAIVLFGSLFAFSIAKEEATKLGTVIGIDLGTTYSCVGVYKNGHVEIIANDQGNRITPSWVAFTDGERLIGEAAKNQAAVNPERTIFDVKRLIGRKFDDKEVQRDKKLVPYEIVNKDGKPYIQVKIKDGETKVFSPEEISAMILTKMKETAEAYLGKKIKDAVVTVPAYFNDAQRQATKDAGVIAGLNVARIINEPTAAAIAYGLDKKGGEKNILVFDLGGGTFDVSILTIDNGVFEVLATNGDTHLGGEDFDQRIMEYFIKLIKKKHGKDISKDNRALGKLRREAERAKRALSSQHQVRVEIESLFDGVDFSEPLTRARFEELNNDLFRKTMGPVKKAMEDAGLEKNQIDEIVLVGGSTRIPKVQQLLKDYFDGKEPNKGVNPDEAVAYGAAVQGGILSGEGGDETKDILLLDVAPLTLGIETVGGVMTKLIPRNTVIPTKKSQVFTTYQDQQTTVTISVFEGERSLTKDCRLLGKFDLTGIAPAPRGTPQIEVTFEVDANGILNVKAEDKASGKSEKITITNDKGRLSQEEIERMVKEAEEFAEEDKKVKERIDARNSLETYVYNMRNQINDKDKLADKLESDEKEKIETATKEALEWLDDNQSAEKEDYDEKLKEVEAVCNPIITAVYQRSGGAPGGASEESNEDDDSHDEL

>*N.tabacum* BiP 5 [SWISS-PORT: Q03684]

MAGGAWNRRTSLIVFGIVLFGCLFAFSIATEEATKLGTVIGIDLGTTYSCVGVYKNGHVEIIANDQGNRITPSWVAFTDGERLIGEAAKNLAAVNPERTVFDVKRLIGRKFDDKEVQRDMKLVPYKIVNKDGKPYIQVKIKDGETKIFSPEEISAMILTKMKETAEAYLGKKIKDAVVTVPAYFNDAQRQATKDAGVIAGLNVARIINEPTAAAIAYGLDKKGGEKNILVFDLGGGTFDVSILTIDNGVFEVLSTNGDTHLGGEDFDQRIMEYFIKLIKKKHGKDISKDNRALGKLRREAERAKRALSSQHQVRVEIESLFDGVDFSEPLTRARFEELNNDLFRKTMGPVKKAMDDAGLEKTQIDEIVLVGGSTRIPKVQQLLKDYFDGKEPNKGVNPDEAVAYGAAVQGGILSGEGGDETKDILLLDVAPLTLGIETVGGVMTKLIPRNTVIPTKKSQVFTTYQDQQTTVTIQVFEGERSLTKDCRLLGKFDLTGIAPAPRGTPQIEVTFEVDANGILNVKAEDKASGKSEKITITNDKGRLSQEEIERMVKEAEEFAEEDKKVKERIDARNSLETYVYNMRNQINDKDKLADKLESDEKEKIETATKEALEWLDDNQSAEKEDYEEKLKEVEAVCNPIITAVYQKSGGAPGGESGASEDDDHDEL

>*V.vinifera* BiP1[GenBank: CBI34546]

MESSWRRRGSLIVAAIVCFGFLAAISIAKEEATKLGTVIGIDLGTTYSCVGVYKNGHVEIIANDQGNRITPSWVAFTDSERLIGEAAKNQAAVNAERTIFDVKRLIGRKFEDKEVQKDMKLVPYNIVNKDGKPYIQVKIKDGETKVFSPEEISAMILTKMKETAEAFLGKKIKDAVVTVPAYFNDAQRQATKDAGVIAGLNVARIINEPTAAAIAYGLDKKGGEKNILVFDLGGGTFDVSILTIDNGVFEVLATNGDTHLGGEDFDQRIMEYFIKLIKKKHGKDISKDNRALGKLRREAERAKRALSSQHQVRVEIESLYDGLDFSEPLTRARFEELNNDLFRKTMGPVKKAMEDAGLEKRQIDEIVLVGGSTRIPKVQQLLKDYFDGKEPNKGVNPDEAVAFGAAVQGSILSGEGGDETKDILLLDVAPLTLGIETVGGVMTKLIPRNTVIPTKKSQVFTTYQDQQTTVSIQVFEGERSLTKDCRQLGKFDLNGIPPAPRGTPQIEVTFEVDANGILNVKAEDKGTGKSEKITITNDKGRLSQEEIDRMVREAEEFAEEDKKVKEKIDEVEAVCNPIITAVYQRSGGAPGAGSDGGEDDDSHDEL

>*V.vinifera* BiP2 [GenBank: CBI23034]

MYGAWGLRASSVVLGILLVGCLVAISIAKEESNKLGTVIGIDLGTTYSCVGVYKNGHVEIIANDQGNRITPSWVAFTDTERLIGEAAKNQAAVNAERTVFDVKRLIGRKFDDKEVQKDMKLFPFKIVNKDGKPYIQVKIKDGETKVFSPEEISAMILTKMKETAEAFLGKTIKDAVVTVPAYFNDAQRQATKDAGIIAGLNVARIINEPTAAAIAYGLDKKGGEKNILVFDLGGGTFDVSILTIDNGVFEVLATNGDTHLGGEDFDQRIMEYFIKLIKKKHGKDISKDNRAIGKLRRESERAKRALSSQHQVRVEIESLYDGLDFSEPLTRARFEELNNDLFRKTMGPVKKAMEDAGLEKRQIDEIVLVGGSTRIPKVQQLLKEYFDGKEPNKGVNPDEAVAYGAAVQGSILSGEGGDETKDILLLDVAPLTLGIETVGGVMTKLIPRNTVIPTKKSQVFTTYQDQQTTVSIQVFEGERSLTKDCRELGKFDLSGIAPAPRGTPQIEVTFEVDANGILNVKAEDKASGKSEKITITNDKEEDKKIKEKIDARNSLESYVYNMKTQINDKDKLADKLESEEKEKIETAVKEALEWLDDNQNAETEDYQEKLKEVEAVCNPIITAVYQRSGGAPGGSSDAGEDEDSHDEL

>*P.falciparum* [EMBL: X69121]

MKQIRPYILLLIVSLLKFISAVDSNIEGPVIGIDLGTTYSCVGVFKNGRVEILNNELGNRITPSYVSFVDGERKVGEAAKLEATLHPTQTVFDVKRLIGRKFDDQEVVKDRSLLPYEIVNNQGKPNIKVQIKDKDTTFAPEQISAMVLEKMKEIAQSFLGKPVKNAVVTVPAYFNDAQRQATKDAGTIAGLNIVRIINEPTAAALAYGLDKKEETSILVYDLGGGTFDVSILVIDNGVFEVYATAGNTHLGGEDFDQRVMDYFIKMFKKKNNIDLRTDKRAIQKLRKEVEIAKRNLSVVHSTQIEIEDILEGHNFSETLTRAKFEELNDDLFRETLEPVKKVLDDAKYEKSKIDEIVLVGGSTRIPKIQQIIKEFFNGKEPNRGINPDEAVAYGAAIQAGIILGEELQDVVLLDVTPLTLGIETVGGIMTQLIKRNTVIPTKKSQTFSTYQDNQPAVLIQVFEGERALTKDNHLLGKFELSGIPPAQRGVPKIEVTFTVDKNGILHVEAEDKGTGKSRGITITNDKGRLSKEQIEKMINDAEKFADEDKNLREKVEAKNNLDNYIQPIIVKLYGQPGGPSPQPSGDEDVDSDEL

>*H.sapiens* [SWISS-PORT: P11021]

MKLSLVAAMLLLLSAARAEEEDKKEDVGTVVGIDLGTTYSCVGVFKNGRVEIIANDQGNRITPSYVAFTPEGERLIGDAAKNQLTSNPENTVFDAKRLIGRTWNDPSVQQDIKFLPFKVVEKKTKPYIQVDIGGGQTKTFAPEEISAMVLTKMKETAEAYLGKKVTHAVVTVPAYFNDAQRQATKDAGTIAGLNVMRIINEPTAAAIAYGLDKREGEKNILVFDLGGGTFDVSLLTIDNGVFEVVATNGDTHLGGEDFDQRVMEHFIKLYKKKTGKDVRKDNRAVQKLRREVEKAKALSSQHQARIEIESFYEGEDFSETLTRAKFEELNMDLFRSTMKPVQKVLEDSDLKKSDIDEIVLVGGSTRIPKIQQLVKEFFNGKEPSRGINPDEAVAYGAAVQAGVLSGDQDTGDLVLLHVCPLTLGIETVGGVMTKLIPSNTVVPTKNSQIFSTASDNQPTVTIKVYEGERPLTKDNHLLGTFDLTGIPPAPRGVPQIEVTFEIDVNGILRVTAEDKGTGNKNKITITNDQNRLTPEEIERMVNDAEKFAEEDKKLKERIDTRNELESYAYSLKNQIGDKEKLGGKLSSEDKETMEKAVEEKIEWLESHQDADIEDFKAKKKELEEIVQPIISKLYGSAGPPPTGEEDTAEKDEL

>*M.auratus* [SWISS-PORT: P07823]

MKFPMVAAALLLLCAVRAEEEDKKEDVGTVVGIDLGTTYSCVGVFKNGRVEIIANDQGNRITPSYVAFTPEGERLIGDAAKNQLTSNPENTVFDAKRLIGRTWNDPSVQQDIKFLPFKVVEKKTKPYIQVDIGGGQTKTFAPEEISAMVLTKMKETAEAYLGKKVTHAVVTVPAYFNDAQRQATKDAGTIAGLNVMRIINEPTAAAIAYGLDKREGEKNILVFDLGGGTFDVSLLTIDNGVFEVVATNGDTHLGGEDFDQRVMEHFIKLYKKKTGKDVRKDNRAVQKLRREVEKAKRALSSQHQARIEIESFFEGEDFSETLTRAKFEELNMDLFRSTMKPVQKVLEDSDLKKSDIDEIVLVGGSTRIPKIQQLVKEFFNGKEPSRGINPDEAVAYGAAVQAGVLSGDQDTGDLVLLDVCPLTLGIETVGGVMTKLIPRNTVVPTKKSQIFSTASDNQPTVTIKVYEGERPLTKDNHLLGTFDLTGIPPAPRGVPQIEVTFEIDVNGILRVTAEDKGTGNKNKITITNDQNRLTPEEIERMVNDAEKFAEEDKKLKERIDTRNELESYAYSLKNQIGDKEKLGGKLSSEDKETMEKAVEEKIEWLESHQDADIEDFKAKKKELEEIVQPIISKLYGSAGPPPTGEEDTSEKDEL

>*M.musculus* [SWISS-PORT: P20029]

MMKFTVVAAALLLLGAVRAEEEDKKEDVGTVVGIDLGTTYSCVGVFKNGRVEIIANDQGNRITPSYVAFTPEGERLIGDAAKNQLTSNPENTVFDAKRLIGRTWNDPSVQQDIKFLPFKVVEKKTKPYIQVDIGGGQTKTFAPEEISAMVLTKMKETAEAYLGKKVTHAVVTVPAYFNDAQRQATKDAGTIAGLNVMRIINEPTAAAIAYGLDKREGEKNILVFDLGGGTFDVSLLTIDNGVFEVVATNGDTHLGGEDFDQRVMEHFIKLYKKKTGKDVRKDNRAVQKLRREVEKAKRALSSQHQARIEIESFFEGEDFSETLTRAKFEELNMDLFRSTMKPVQKVLEDSDLKKSDIDEIVLVGGSTRIPKIQQLVKEFFNGKEPSRGINPDEAVAYGAAVQAGVLSGDQDTGDLVLLDVCPLTLGIETVGGVMTKLIPRNTVVPTKKSQIFSTASDNQPTVTIKVYEGERPLTKDNHLLGTFDLTGIPPAPRGVPQIEVTFEIDVNGILRVTAEDKGTGNKNKITITNDQNRLTPEEIERMVNDAEKFAEEDKKLKERIDTRNELESYAYSLKNQIGDKEKLGGKLSSEDKETMEKAVEEKIEWLESHQDADIEDFKAKKKELEEIVQPIISKLYGSGGPPPTGEEDTSEKDEL

>*R.norvegicus* [SWISS-PORT: P06761]

MKFTVVAAALLLLCAVRAEEEDKKEDVGTVVGIDLGTTYSCVGVFKNGRVEIIANDQGNRITPSYVAFTPEGERLIGDAAKNQLTSNPENTVFDAKRLIGRTWNDPSVQQDIKFLPFKVVEKKTKPYIQVDIGGGQTKTFAPEEISAMVLTKMKETAEAYLGKKVTHAVVTVPAYFNDAQRQATKDAGTIAGLNVMRIINEPTAAAIAYGLDKREGEKNILVFDLGGGTFDVSLLTIDNGVFEVVATNGDTHLGGEDFDQRVMEHFIKLYKKKTGKDVRKDNRAVQKLRREVEKAKRALSSQHQARIEIESFFEGEDFSETLTRAKFEELNMDLFRSTMKPVQKVLEDSDLKKSDIDEIVLVGGSTRIPKIQQLVKEFFNGKEPSRGINPDEAVAYGAAVQAGVLSGDQDTGDLVLLDVCPLTLGIETVGGVMTKLIPRNTVVPTKKSQIFSTASDNQPTVTIKVYEGERPLTKDNHLLGTFDLTGIPPAPRGVPQIEVTFEIDVNGILRVTAEDKGTGNKNKITITNDQNRLTPEEIERMVNDAEKFAEEDKKLKERIDTRNELESYAYSLKNQIGDKEKLGGKLSPEDKETMEKAVEEKIEWLESHQDADIEDFKAKKKELEEIVQPIISKLYGSGGPPPTGEEDTSEKDEL

>*X.laevis* [GenBank: U62807]

MVTMKLFALVLLVSASVFASDDDDKKDDIGTVVGIDLGTTYSCVGVFKNGRVEIIANDQGNRITPSYVAFTPEGERLIGDAAKNQLTSNPENTVFDAKRLIGRTWNDPSVQQDIKYLPFKVIEKKTKPYIEVDIGDQMKTFAPEEISAMVLVKMKETAEAYLGRKVTHAVVTVPAYFNDAQRQATKDAGTIAGLNVMRIINEPTAAAIAYGLDKKEGEKNILVFDLGGGTFDVSLLTIDNGVFEVVATNGDTHLGGEDFDQRVMEHFIKLYKKKTGKDVRADKRAVQKLRREVEKAKRALSAQHQSRIEIESFFEGEDFSETLTRAKFEELNMDLFRSTMKPVQKVLDDSDLKKSDIDEIVLVGGSTRIPKIQQLVKELFNGKEPSRGINPDEAVAYGAAVQAGVSGDQDTGDLVLLDVCPLTLGIETVGGVMTKLIPRNTVVPTKKSQIFSTASDNQPTVTIKVYEGERPLTKDNHLLGTFDLTGIPPAPRGVPQIEVTFEIDVNGILRVTAEDKGTGNKNKITITNDQNRLTPEEIERMVTDAEKFAEEDKKLKERIDTRNELESYAYSLKNQIGDKEKLGGKLSSEDKETIEKAVEEKIEWLESHQDADIEDFKAKKKELEEIVQPIVGKLYGGAGAPPPEGAEGAEETEKDEL

>*T.brucei* [GenBank: L14477]

MSRMWLTTAAVFLTVTVAAVSAAPESGGKVEAPCVGIDLGTTYSVVGVWQKGDVHIIPNEMGNRITPSVVAFTDTERLIGDGAKNQLPQNPHNTIYTIKRLIGRKYTDAAVQADKKLLSYEVIADRDGKPKVQVMVGGKKKQFTPEEISAMVLQKMKEIAETYLGEKVKNAVVTVPAYFNDAQRQSTKDAGTIAGLNVVRIINEPTAAAIAYGLNKAGEKNILVFDLGGGTFDVSLLTIDEGFFEVVATNGDTHLGGEDFDNNMMRHFVDMLKKKKNVDISKDQKALARLRKACEAAKRQLSSHPEARVEVDSLTEGFDFSEKITRAKFEELNMDLFKGTLVPVQRVLEDAKLKKSDIHEIVLVGGSTRVPKVQQLISDFFGGKELNRGINPDEAVAYGAAVQAAVLTGESEVGGRVVLVDVIPLSLGIETVGGVMTKLIERNTQIPTKKSQVFSTHADNQPGVLIQVYEGERQLTKDNRLLGKFELSGIPPAARGVPQIEVTFDVDENSILQVSAMDKSSGKKEEITITNDKGRLSEEEIERMVREAAEFEDEDRKVRERVDARNSLESVAYSLRNQVNDKDKLGGKLDPNDKAAVETAVAEAIRFLDENPNAEKEEYKTALETLQSVTNPIIQKTYQSAGGGDKPQPMDDL

>*M.pumila* [GenBank: AAW55475]

MAGSWRARGSLIVLAIVTFGCLFAFSIAKEEATKLGTVIGIDLGTTYSCVGVYKNGHVEIIANDQGNRITPSWVAFSDSERLIGEAAKNQAAVNPERTVFDVKRLIGRKFEDKEVQKDMKLVPYKIVNRDGKPYIQVRIKDGETKVFSPEEVSAMILTKMKETAEAFLGKKIKDAVVTVPAYFNDAQRQATKDAGVIAGLNVARIINEPTAAAIAYGLDKKGGEKNILVFDLGGGTFDVSILTIDNGVFEVLSTNGDTHLGGEDFDQRIMEYFIKLIKKKHGKDISKDNRALGKLRREAERAKRALSSQHQVRVEIESLFDGVDFSEPLTRARFEELNNDLFRKTMGPVKKAMDDAGLEKRQIDEIVLVGGSTRIPKVQQLLKDYFDGKEPNKGANPDEAVAYGAAVQGSILSGEGGEETKDILLLDVAPLTLGIETVGGVMTKLIPRNTVIPTKKSQVFTTYQDQQTTVSIQVFEGERSLTKDCRNLGKFDLTGIPPAPRGTPQIEVTFEVDANGVLNVRAEDKGTGKAEKITITNDKGRLSQEEIERMVKEAEEFAEEDKKVKERIDARNSLETYVYNMKNQINDKDKLADKLESDEKEKIETATKEALEWLDDNQTAEKEDYDEKLKEVEAVCNPIISAVYQRSGGAPGGAGASEEDDESHDEL

>*G.hirsutum* [GenBank: ACJ11746]

MARSWRASGSLVALAIVLSGCFFAISIAKEEAAKLGTVIGIDLGTTYSCVGVYKNGHVEIIANDQGNRITPSWVAFTDSERLIGEAAKNQAAVNAERTIFDVKRLIGRKFEDKEVQRDMKLVPYKIVNKDGKPYIQVKIKDGETKVFSPEEISAMVLTKMKETAEAFLGKKIKDAVVTVPAYFNDAQRQATKDAGIIAGLNVARIINEPTAAAIAYGLDKKGGEKNILVFDLGGGTFDVSILTIDNGVFEVLSTNGDTHLGGEDFDQRIMEYFIKLIKKKHGKDISKDNRALGKLRREAERAKRALSSQHQVRVEIESLFDGVDFSEPLTRARFEELNNDLFRKTMGPVKKAMEDAGLQKSQIDEIVLVGGSTRIPKVQQLLKDYFDGKEPNKGVNPDEAVAYGAAVQGGILSGEGGDETKDILLLDVAPLTLGIETVGGVMTKLIPRNTVIPTKKSQVFTTYQDQQTTVSIQVFEGERSLTKDCRLLGKFDLTGIPPAPRGTPQIEVTFEVDANGILNVKAEDKGTGKSEKITITNDKGRLSQEEIERMVREAEEFAEEDKKVKERIDARNSLETYIYNMKNQINDKDKLADKLESDEKEKVETAVKEALEWLDDNQSAEKEDYEEKLKEVEAVCNPIITAVYQRSGGAPGGGSTEEDDDSHDEL

>*I.tinctoria* [GenBank: AAZ95244]

MARSFGANGTVVLAIIFFGCLFALCTAKEEATKLGTVIGIDLGTTYSCVGVYKNGHVEIIANDQGNRITPSWVGFTDSERLIGEAAKNQAAVNPERTIFDVKRLIGRKFEDKEVQKDRKLVPYQIVNKDGKPYIQVKIKDGETKVFSPEEISAMILTKMKETAEAYLGKKIKDAVVTVPAYFNDAQRQATKDAGVIAGLNVARIINEPTAAAIAYGLDKKGGEKNILVFDLGGGTFDVSVLTIDNGVFEVLSTNGDTHLGGEDFDHRIMEYFIKLIKKKHQKDISKDNKALGKLRRECERAKRALSSQHQVRVEIESLFDGVDFSEPLTRARFEELNNDLFRKTMGPVKKAMDDAGLQKSQIDEIVLVGGSTRIPKVQQLLKDFFEGKEPNKGVNPDEAVAYGAAVQGGILSGEGGDETKDILLLDVAPLTLGIETVGGVMTKLIPRNTVIPTKKSQVFTTYQDQQTTVSIQVFEGERSLTKDCRLLGKFDLNGIPPAPRGTPQIEVTFEVDANGILNVKAEDKASGKSEKITITNEKGRLSQEEIDRMVKEAEEFAEEDKKVKERIDARNSLETYVYNMKNQVSDKDKLADKLEADEKEKIEAATKEALEWLDENQNSEKEDYEEKLKEVEAVCNPIITAVYQRSGGAPGAGGESAPEEEDESHDEL

>*N.crassa* [EMBL: Y09011]

MEHRTRSWALGLSILGFFALLFSAGFVQQAHANDTEAMGTVIGIDLGTTYSCVGVMQKGKVEILVNDQGNRITPSYVAFTDEERLVGDAAKNQAAANPHRTIFDIKRLIGRKFSDKDVQNDIKHFPNKVVSKDDKPVVKVEVKGEEKTFTPEEISAMILGKMKETAEGYLGKKVTHAVVTVPAYFNDNQRQATKDAGMIAGLNVLRIVNEPTAAAIAYGLDKTGEERQIIVYDLGGGTFDVSLLSIEQGVFEVLSTAGDTHLGGEDFDQRIINHFAKLFNKKHGVDVTKDAKAMGKLKREAEKAKRTLSSQMSTRIEIEAFYDGKDFSETLTRAKFEELNNDLFKKTLKPVEQVLKDAKVSKSEIDDIVLVGGSTRIPKVQALIEEFFNGKKASKGINPDEAVAFGAAVQAGVLSGEEGTEDIVLMDVNPLTLGIETTGGVMTKLIPRNTPIPTRKSQIFSTAADNQPVVLIQVYEGERSMTKDNNLLGKFELTGIPPAPRGVPQIEVSFELDANGILKVSAHDKGNGKAESITITNEKGRLTQEEIDRMVAEAEKYAEEDKATRERIEARNGLENYAFSLKNQVNDEDGLGGKIDEEDKETILDAVKEAQDWLEENDAATASAEDFDEQKEKLSNVAYPITSKLYSQGGAGDDEPAGHDEL

>*S.cerevisiae* [SWISS-PORT: P16474]

MFFNRLSAGKLLVPLSVVLYALFVVILPLQNSFHSSNVLVRGADDVENYGTVIGIDLGTTYSCVAVMKNGKTEILANEQGNRITPSYVAFTDDERLIGDAAKNQVAANPQNTIFDIKRLIGLKYNDRSVQKDIKHLPFNVVNKDGKPAVEVSVKGEKKVFTPEEISGMILGKMKQIAEDYLGTKVTHAVVTVPAYFNDAQRQATKDAGTIAGLNVLRIVNEPTAAAIAYGLDKSDKEHQIIVYDLGGGTFDVSLLSIENGVFEVQATSGDTHLGGEDFDYKIVRQLIKAFKKKHGIDVSDNNKALAKLKREAEKAKRALSSQMSTRIEIDSFVDGIDLSETLTRAKFEELNLDLFKKTLKPVEKVLQDSGLEKKDVDDIVLVGGSTRIPKVQQLLESYFDGKKASKGINPDEAVAYGAAVQAGVLSGEEGVEDIVLLDVNALTLGIETTGGVMTPLIKRNTAIPTKKSQIFSTAVDNQPTVMIKVYEGERAMSKDNNLLGKFELTGIPPAPRGVPQIEVTFALDANGILKVSATDKGTGKSESITITNDKGRLTQEEIDRMVEEAEKFASEDASIKAKVESRNKLENYAHSLKNQVNGDLGEKLEEEDKETLLDAANDVLEWLDDNFETAIAEDFDEKFESLSKVAYPITSKLYGGADGS

GAADYDDEDEDDDGDYFEHDEL

>*C.elegans* [GenBank: U56965]

MKTLFLLGMIAITAVSIYCKEEEKTEKKETKYETIIGIDLGTTYSCVGVYKNGRVEIIANDQGNRITPSYVAFSGDQGDRLIGDAAKNQLTINPENTIFDAKRLIGRDYNDKTVQADIKHWPFKVIDKSNKPSVEVKVGSDNKQFTPEEVSAMVLVKMKEIAESYLGKEVKNAVVTVPAYFNDAQRQATKDAGTIAGLNVVRIINEPTAAAIAYGLDKKDGERNILVFDLGGGTFDVSMLTIDNGVFEVLATNGDTHLGGEDFDQRVMEYFIKLYKKKSGKDLRKDKRAVQKLRREVEKAKRALSTQHQTKVEIESLFDGEDFSETLTRAKFEELNMDLFRATLKPVQKVLEDSDLKKDDVHEIVLVGGSTRIPKVQQLIKEFFNGKEPSRGINPDEAVAYGAAVQGGVISGEEDTGEIVLLDVNPLTMGIETVGGVMTKLIGRNTVIPTKKSQVFSTAADNQPTVTIQVFEGERPMTKDNHQLGKFDLTGLPPAPRGVPQIEVTFEIDVNGILHVTAEDKGTGNKNKITITNDQNRLSPEDIEAMINDAEKFAEDDKKVKDKAEARNELESYAYNLKNQIEDKEKLGGKLDEDDKKTIEEAVEEAISWLGSNAEASAEELKEQKKDLESKVQPIVSKLYKDAGAGERRPQKRDLDDKDEL

>*P.trichocarpa* [GenBank: NP_002299448]

MAGTWTARGVVVSAIILFGCLFAISIAKEEATKLGTVIGIDLGTTYSCVGVYKNGHVEIIANDQGNRITPSWVAFTDSERLIGEAAKNQAAVNAERTIFDVKRLIGRKFEDKEVQKDMKLFPYKIVNKDGKPYIQVKIKDGETKVFSPEEISAMILTKMKETAEAFLGKKIKDAVVTVPAYFNDAQRQATKDAGIIAGLNVARIINEPTAAAIAYGLDKKGGEKNILVFDLGGGTFDVSVLTIDNGVFEVLSTNGDTHLGGEDFDQRIMEYFIKLIKKKHGKDISKDNRALGKLRRECERAKRALSSQHQVRVEIESLYDGMDFSEPLTRARFEELNNDLFRKTMGPVKKAMEDAGLEKNQIDEIVLVGGSTRIPKVQQLLKDYFDGKEPNKGVNPDEAVAFGAAVQGGILSGEGGDETKDILLLDVAPLTLGIETVGGVMTKLIPRNTVIPTKKSQVFTTYQDQQTTVSIQVFEGERSLTKDCRLLGKFDLTGVPPAPRGTPQIEVTFEVDANGILNVKAEDKGTGKSEKITITNDKGRLSQEEIERMVREAEEFAEEDKMVKERIDARNSLETYVYNMKNQINDKDKLADKLESDEKEKIETATKETLEWLDDNQNAEKEDYEEKLKEVEAVCNPIITAVYQRSGGAPGGGSAEDPEDDSHDEL

>*C.arietinum* [GenBank: XP_004503582]

MAGSWKCGSLFVLAIISFGCLFAISIAKEEGTKLGTVIGIDLGTTYSCVGVYKNGHVEIIANDQGNRITPSWVAFTDSERLIGEAAKNLAAVNPERTIFDVKRLIGRKFEDKEVQRDMKLVPYKIVNKDGKPYIQVKIKDGETKVFSPEEISAMILTKMKETAEAFLGKTIRDAVVTVPAYFNDAQRQATKDAGVIAGLNVARIINEPTAAAIAYGLDKKGGEKNILVFDLGGGTFDVSILTIDNGVFEVLSTNGDTHLGGEDFDQRIMEYFIKLIKKKYGKDISKDNRALGKLRRESERAKRALSSQHQIRVEIESLFDGVDFSESLTRARFEELNNDLFRKTMGPVKKAMDDAGLQKNQIDEIVLVGGSTRIPKVQQLLKDYFDGKEPNKGVNPDEAVAFGAAVQGSILSGEGGEETKDILLLDVAPLTLGIETVGGVMTKLIPRNTVIPTKKSQVFTTYQDQQTTVSIQVFEGERSLTKDCRLLGKFDLSGIPPAPRGTPQIEVTFEVDANGILNVKAEDKGTGKSEKITITNEKGRLSQEEIDRMVREAEEFAEEDKKVKERIDARNGLETYVYNMKNQVNDKDKLADKLESDEKEKIETAVKEALEWLDDNQSVEKEDYEEKLKEVEAVCNPIITAVYQRSGGAPGGDASGEGEEDDSHDEL
