# Supplementary material for: Molecular cloning, phylogenetic analysis, and expression profiling of endoplasmic reticulum molecular chaperone BiP genes from bread wheat (Triticum aestivum L.)
Source: BMC Plant Biol. 2014 Oct 1;14:260. doi: 10.1186/s12870-014-0260-0 (PMC4189733; doi:10.1186/s12870-014-0260-0)

a. Double standard curve and dissolution curve of the RT-PCR of *TaBiP* in different wheat tissues.

TaBiP1

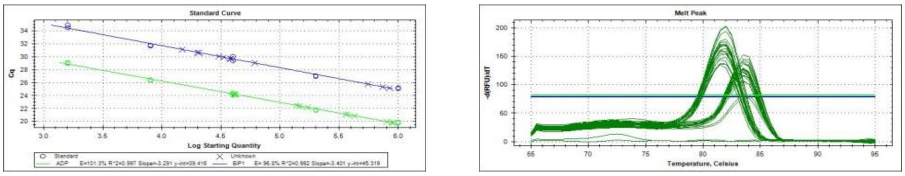

TaBiP2

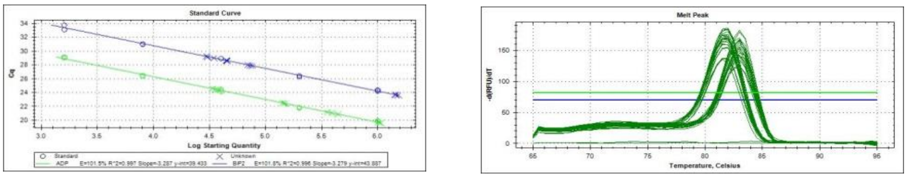

TaBiP3

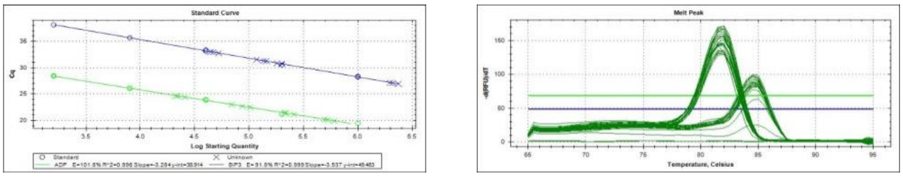

b. Double standard curve and dissolution curve of the RT-PCR of *TaBiP* in different wheat tissues under different abiotic stress.

In root under 20%PEG

TaBiP1

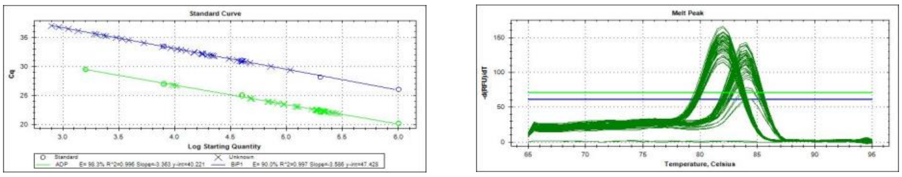

TaBiP2

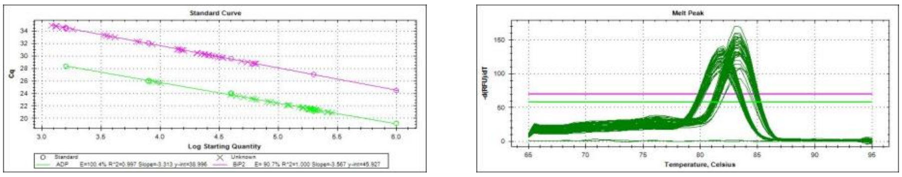

TaBiP3

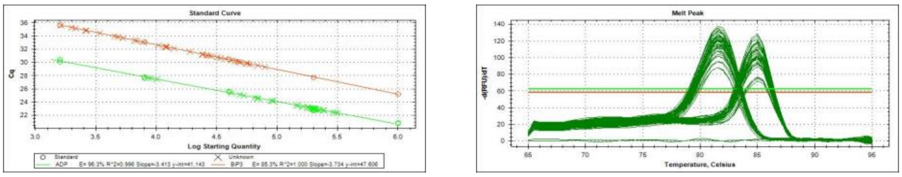

In leaves under 20%PEG

TaBiP1

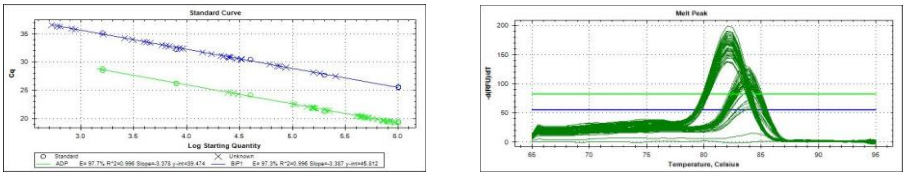

TaBiP2

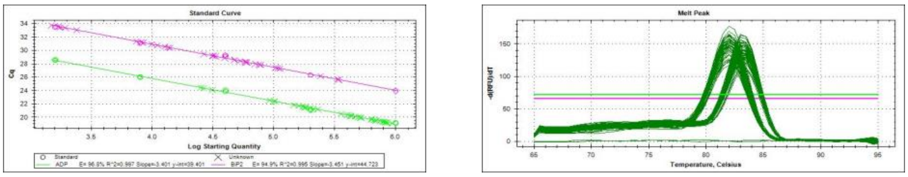

TaBiP3

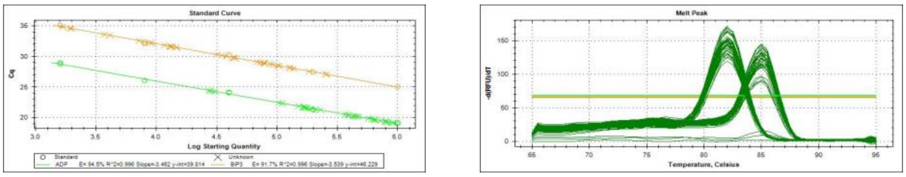

In leaves under different concentration PEG

TaBiP1

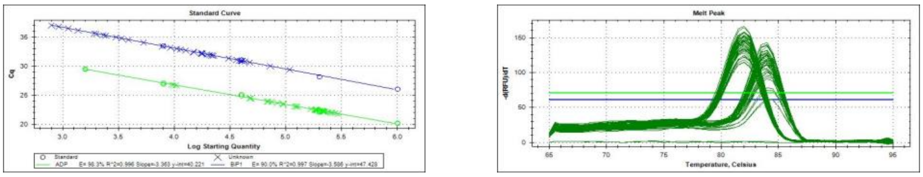

TaBiP2

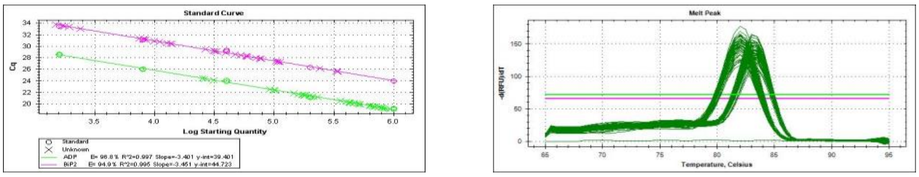

TaBiP3

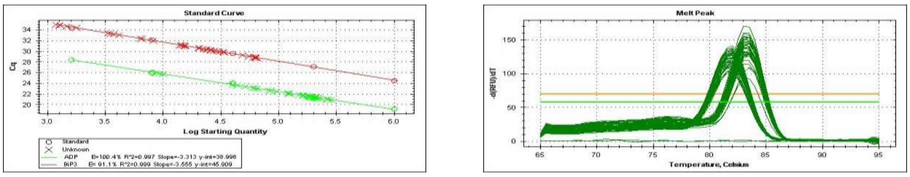

c. Double standard curve and dissolution curve of the RT-PCR of TaBiP in developing seeds and under drought stress

***TaBiP1* of yanyou361CK**

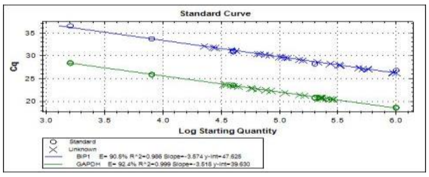

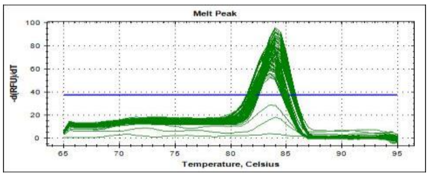

***TaBiP1* of yanyou361GH**

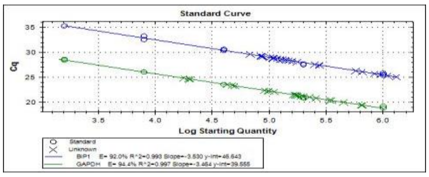

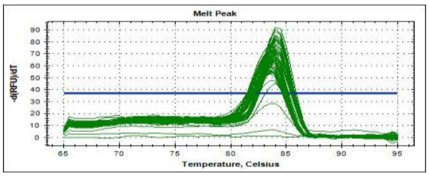

***TaBiP2* of yanyou361CK**

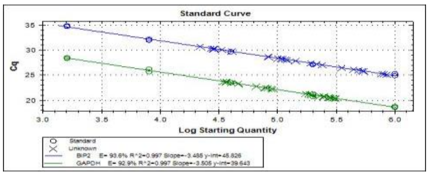

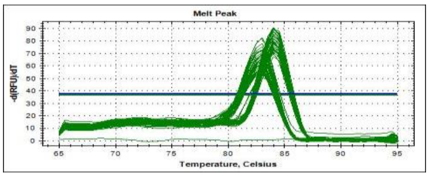

***TaBiP2* of yanyou361GH**

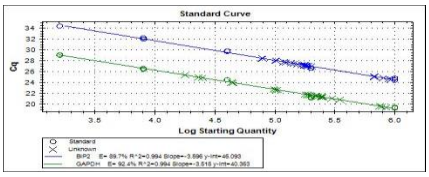

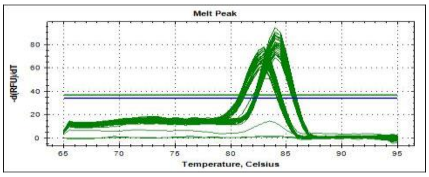

***TaBiP3* of yanyou361CK**

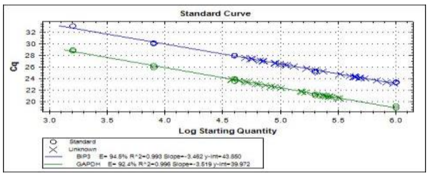

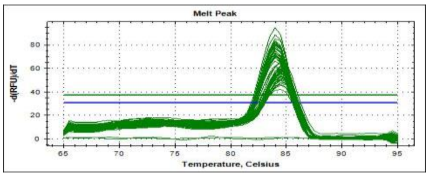

***TaBiP3* of yanyou361GH**

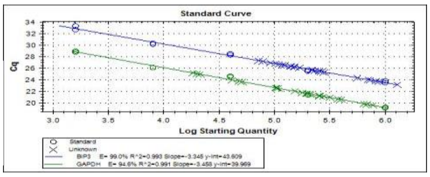

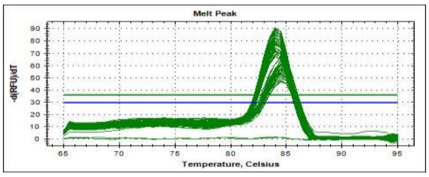

d. Double standard curve and dissolution curve of the RT-PCR of TaBiP in different subunits of developing seeds.

Double standard curve and dissolution curve of the RT-PCR of *TaBiP1* in different subunits of developing seeds.

L03-222

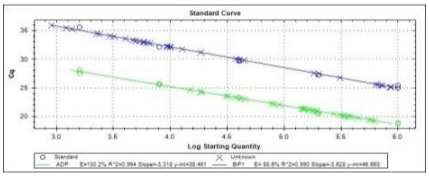

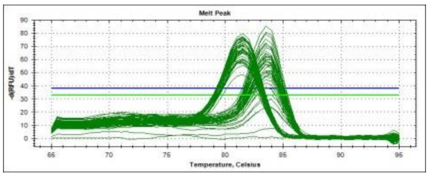

L03-227

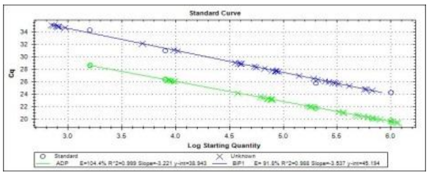

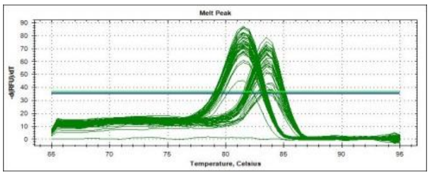

L03-228

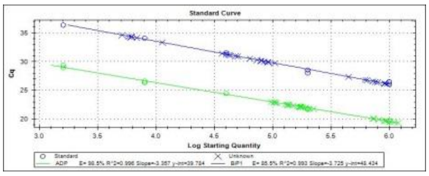

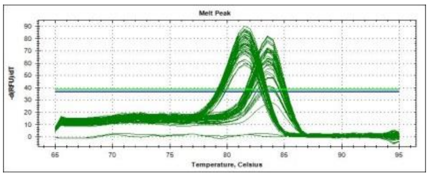

L03-231

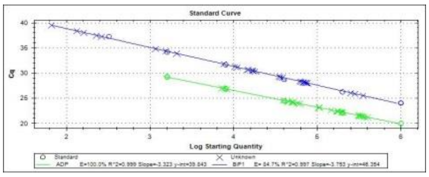

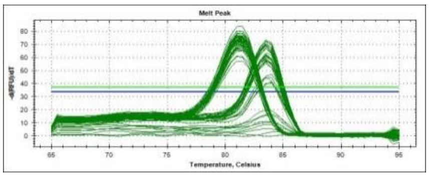

L03-233

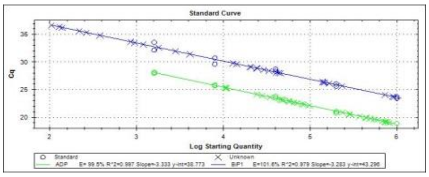

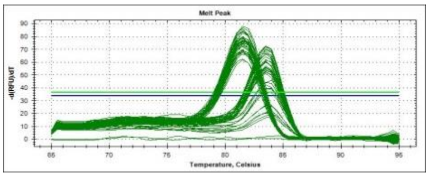

L03-235

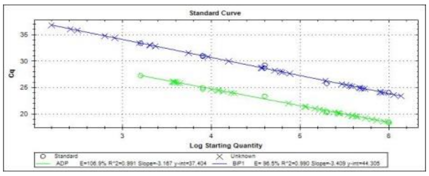

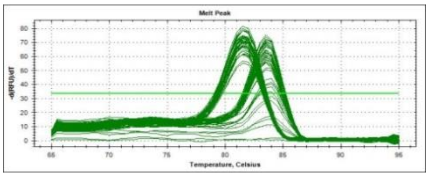

L03-238

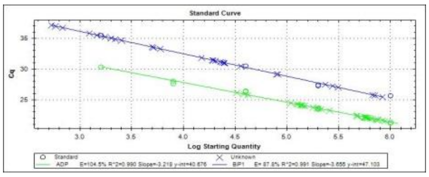

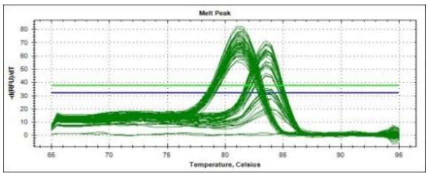

L03-240

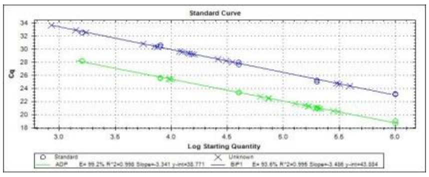

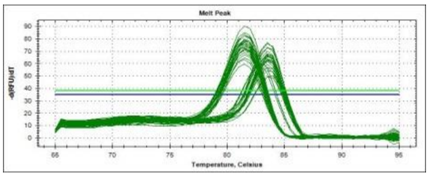

Double standard curve and dissolution curve of the RT-PCR of *TaBiP2* in different subunits of developing seeds.

L03-222

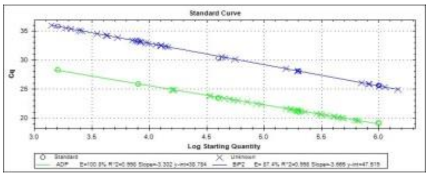

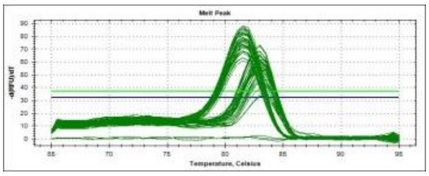

L03-227

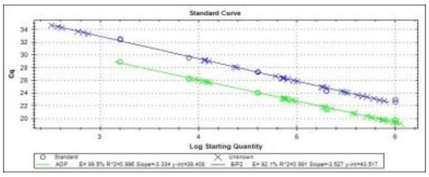

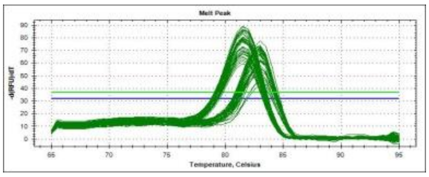

L03-228

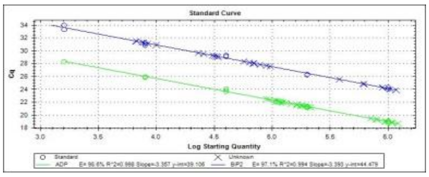

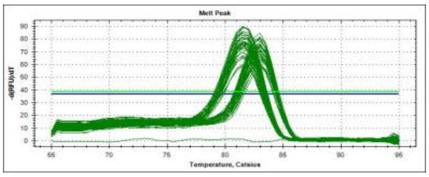

L03-231

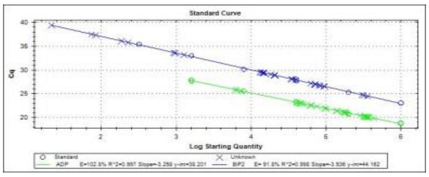

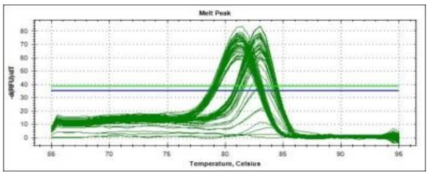

L03-233

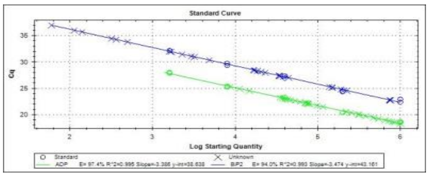

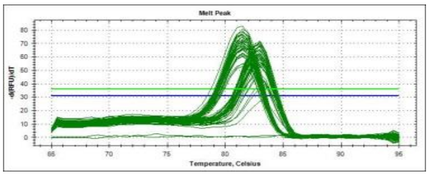

L03-235

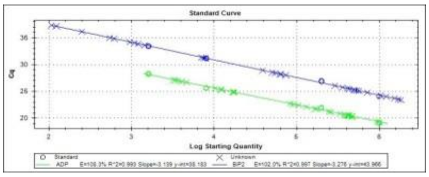

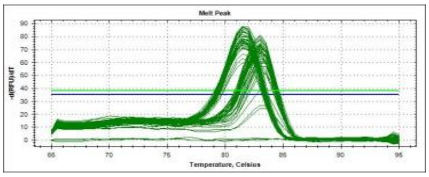

L03-238

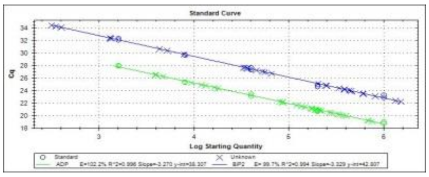

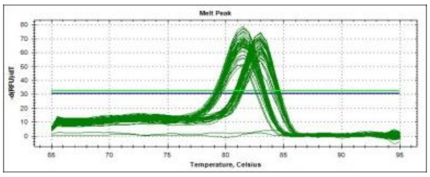

L03-240

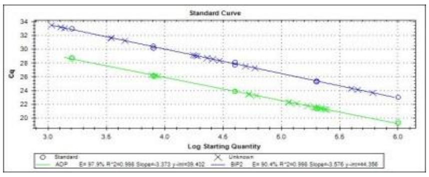

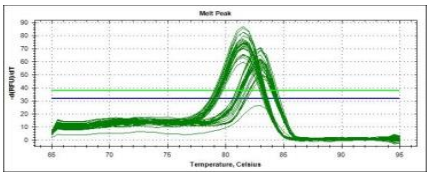

Double standard curve and dissolution curve of the RT-PCR of *TaBiP3* in different subunits of developing seeds.

L03-222

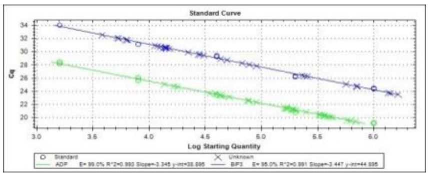

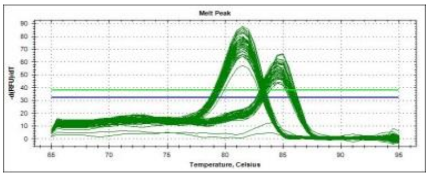

L03-227

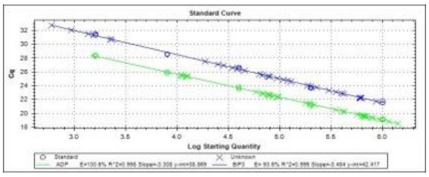

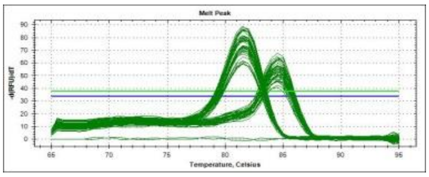

L03-228

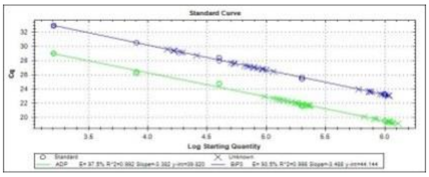

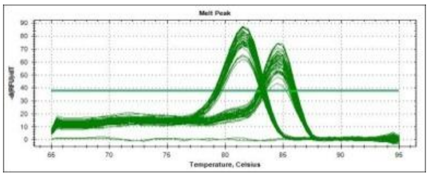

L03-231

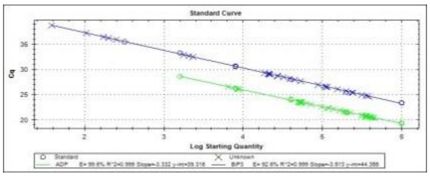

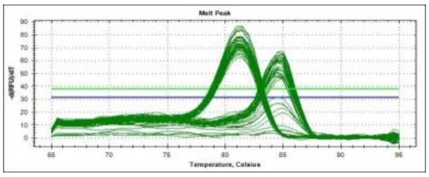

L03-233

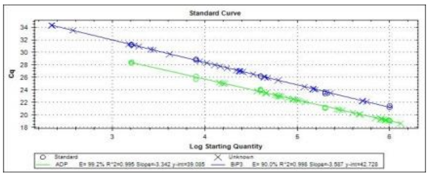

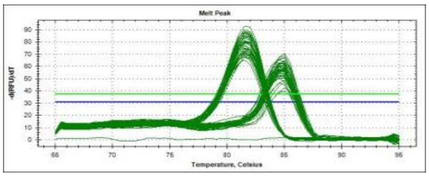

L03-235

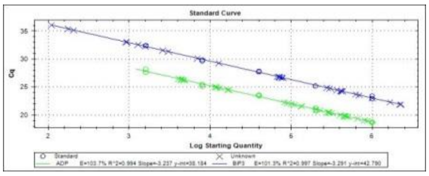

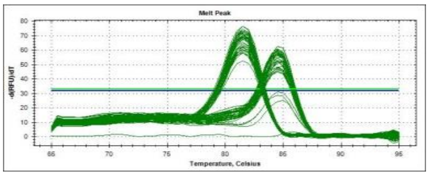

L03-238

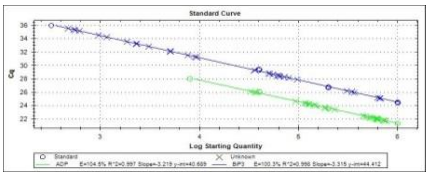

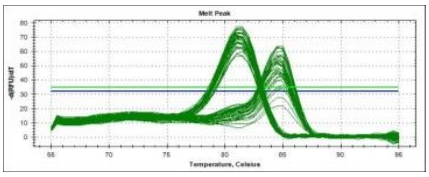

L03-240

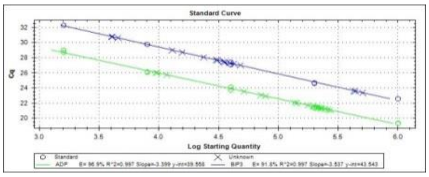

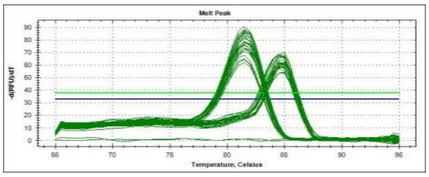

Supplement: Additional file 5: — qRT-PCR optimization design: double standard curves and dissolution curves of TaBiP in wheat tissue, developing seeds, and under stress. [file 12870_2014_260_MOESM5_ESM.pdf]
